# Discovery of indolylpiperazinylpyrimidines with dual-target profiles at adenosine A_2A_ and dopamine D_2_ receptors for Parkinson's disease treatment

**DOI:** 10.1371/journal.pone.0188212

**Published:** 2018-01-05

**Authors:** Yi-Ming Shao, Xiaohua Ma, Priyankar Paira, Aaron Tan, Deron Raymond Herr, Kah Leong Lim, Chee Hoe Ng, Gopalakrishnan Venkatesan, Karl-Norbert Klotz, Stephanie Federico, Giampiero Spalluto, Siew Lee Cheong, Yu Zong Chen, Giorgia Pastorin

**Affiliations:** 1 Department of Pharmacy, National University of Singapore, Singapore; 2 Department of Pharmacology, National University of Singapore, Singapore; 3 Department of Physiology, National University of Singapore, Singapore; 4 National Neuroscience Institute, Singapore; 5 Institut für Pharmakologie, Universität Würzburg, Würzburg, Germany; 6 Dipartimento di Scienze Chimiche e Farmaceutiche, Università degli Studi di Trieste, Trieste, Italy; 7 Department of Pharmaceutical Chemistry, School of Pharmacy, International Medical University, Kuala Lumpur, Malaysia; Centre de Recherche Jean-Pierre Aubert, FRANCE

## Abstract

Parkinson’s disease (PD) is a neurodegenerative disorder characterized by progressive loss of dopaminergic neurons in the *substantia nigra* of the human brain, leading to depletion of dopamine production. Dopamine replacement therapy remains the mainstay for attenuation of PD symptoms. Nonetheless, the potential benefit of current pharmacotherapies is mostly limited by adverse side effects, such as drug-induced dyskinesia, motor fluctuations and psychosis. Non-dopaminergic receptors, such as human A_2A_ adenosine receptors, have emerged as important therapeutic targets in potentiating therapeutic effects and reducing the unwanted side effects. In this study, new chemical entities targeting both human A_2A_ adenosine receptor and dopamine D_2_ receptor were designed and evaluated. Two computational methods, namely support vector machine (SVM) models and Tanimoto similarity-based clustering analysis, were integrated for the identification of compounds containing indole-piperazine-pyrimidine (IPP) scaffold. Subsequent synthesis and testing resulted in compounds **5** and **6**, which acted as human A_2A_ adenosine receptor binders in the radioligand competition assay (*K*_i_ = 8.7–11.2 μM) as well as human dopamine D_2_ receptor binders in the artificial cell membrane assay (EC_50_ = 22.5–40.2 μM). Moreover, compound **5** showed improvement in movement and mitigation of the loss of dopaminergic neurons in *Drosophila* models of PD. Furthermore, *in vitro* toxicity studies on compounds **5** and **6** did not reveal any mutagenicity (up to 100 μM), hepatotoxicity (up to 30 μM) or cardiotoxicity (up to 30 μM).

## Introduction

**Fig 1:**
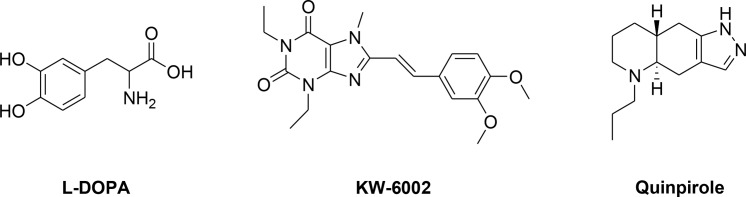
Structures of L-DOPA, KW-6002 and quinpirole.

Parkinson’s disease (PD) is a neurodegenerative disorder characterized by cardinal motor features including tremor, rigidity, bradykinesia and postural instability. It is pathologically associated with loss of dopaminergic neurons in the *substantia nigra* of the human brain, leading to depletion of dopamine production [1]. Over the years, development of pharmacotherapy for PD has been largely focused on improving motor symptoms caused by dopamine deficiency. Among these pharmacotherapies, dopamine replacement therapy represents the major therapeutic approach to alleviate symptoms by restoring dopamine levels. L-DOPA (**Fig 1**), a metabolic precursor of dopamine, remains the most effective dopamine replacement therapy for improving motor deficits. It is able to cross the blood brain barrier (BBB) and is efficiently converted into dopamine by enzymatic decarboxylation [2]. Nevertheless, chronic administration of L-DOPA has been associated with side effects such as dyskinesia, end-of-dose deterioration of function and a switch between mobility and immobility (on/off phenomenon) in the treated patients [3,4]. Hence, L-DOPA is often co-administered with other adjuvant drugs to overcome these side effects. For instance, it is co-administered with dopamine agonists to increase the activity of the dopamine system, or monoamine oxidase B (MAO B) inhibitors and catechol-O-methyltransferase (COMT) inhibitors to prevent the metabolism of dopamine by these enzymes, thus increasing dopamine concentration in the brain. However, these adjuvant drugs are still inadequate in reducing the parkinsonian motor disabilities [5].

In recent years, non-dopaminergic receptors have been identified to play key roles in the pathophysiology of PD. Among these targets, A_2A_ adenosine receptor (A_2A_AR) has gained much attention as an important pharmacological target in counteracting the motor symptoms of PD [6]. It is co-expressed with dopamine D_2_ receptors in striato-pallidal neurons where these receptors are known to form heterodimeric complexes [7,8]. The stimulation of A_2A_AR has been shown to decrease the affinity of D_2_ receptor agonists. Studies have demonstrated that blockade of A_2A_AR through the action of antagonists amplifies the therapeutic effect of L-DOPA and reduces the L-DOPA-induced dyskinesia [8–10]. In addition, A_2A_ antagonists were also reported to exert a neuroprotective effect in which they were able to prevent the onset and development of PD [11]. For these reasons, the combination of hA_2A_AR antagonists and L-DOPA has been investigated for improved efficacy relative to dopamine replacement mono-therapy.

Indeed, tremendous effort has been made towards the development of effective drugs alongside the identification of new therapeutic targets for treatment of PD symptoms. In the past, pharmacotherapies for PD have mostly focused on selective compounds targeting individual proteins (“one compound-one target” approach), particularly the dopamine receptors. Subsequently, discovery of heterodimeric A_2A_ adenosine receptor / dopamine D_2_ receptor complexes in the *striatum* has steered the development of combination therapies (“cocktail drug-multiple targets”) containing an adenosine A_2A_ receptor antagonist and either L-DOPA or a dopamine D_2_ receptor agonist [8,9]. This has been corroborated by marked enhancement of anti-PD activity in 1-methyl-4-phenyl-1,2,3,6-tetrahydropyridine (MPTP)-treated marmosets administered with combination therapy consisting of KW-6002 (an adenosine A_2A_ receptor antagonist, *K*_i_ hA_2A_ = 12 nM, **Fig 1**) and L-DOPA or of KW-6002 and quinpirole (a dopamine D_2_ receptor agonist, *K*_i_ D_2_ = 4.8 nM, **Fig 1**) [12]. Nonetheless, these combination therapies are often associated with side effects arising from drug-drug interactions and varying pharmacokinetic or pharmacodynamic profiles of each component drug. Consequently, “one compound-multiple targets” strategy has emerged as an alternative approach to the management of PD. In such approach, a single drug compound is designed to possess pharmacological activities to multiple targets of interest. The single entity can potentially eliminate side effects derived from interactions amongst drugs in the combination therapies, and improve compliance, especially in elderly patients who are commonly prescribed multiple medications to control the PD.

In our present study, we have successfully employed two virtual screening methods to identify novel scaffolds that simultaneously bind the two receptors—adenosine A_2A_ receptor and dopamine D_2_ receptor—implicated in the PD pathophysiology [13–21].

## Results and discussion

### Design rationale

Adenosine A_2A_ receptor antagonists were identified from the existing literature. Each compound with reported binding or antagonistic activity at A_2A_ receptor therein was manually drawn by ChemDraw [22], and the relevant pharmacological data were noted down on ChemFinder. A total of 1969 adenosine A_2A_ receptor antagonists, of which 1595 had reported binding data (inhibition constant *K*_i_, in most cases), were collected from 69 publications, and were classified into 94 major scaffolds. Most scaffolds were composed of either a xanthine or nitrogen-containing heterocyclic nucleus. Of these selected 1595 compounds reported to bind at A_2A_ receptor subtype, 418 compounds (**S1 Table**) were tested for binding at A_2A_ receptor and further shown to be active in advanced assays relevant to PD, including the ability to block cAMP generation [13], mouse catalepsy model [14], rat catalepsy model [15], as well as Ca^2+^ mobilisation assessment *via* a fluorescence imaging plate reader (FLIPR) assay [16]. Similarly, a total of 810 dopamine D_2_ receptor agonists were collected from 71 publications which focused on identifying agonists for D_2_ receptor. Of these 810 reported compounds, 569 were described as dopamine D_2_ receptor agonists with reported binding data, and were classified into 78 major scaffolds. Of these 569 compounds, 332 (**S1 Table**) were tested for binding at D_2_ receptor and further shown to be active in various advanced assays relevant to PD [17–19]. Moreover, an additional 465 compounds (295 for adenosine A_2A_ receptor and 170 for dopamine D_2_ receptor) were collected from the MDDR database.

**Fig 2:**
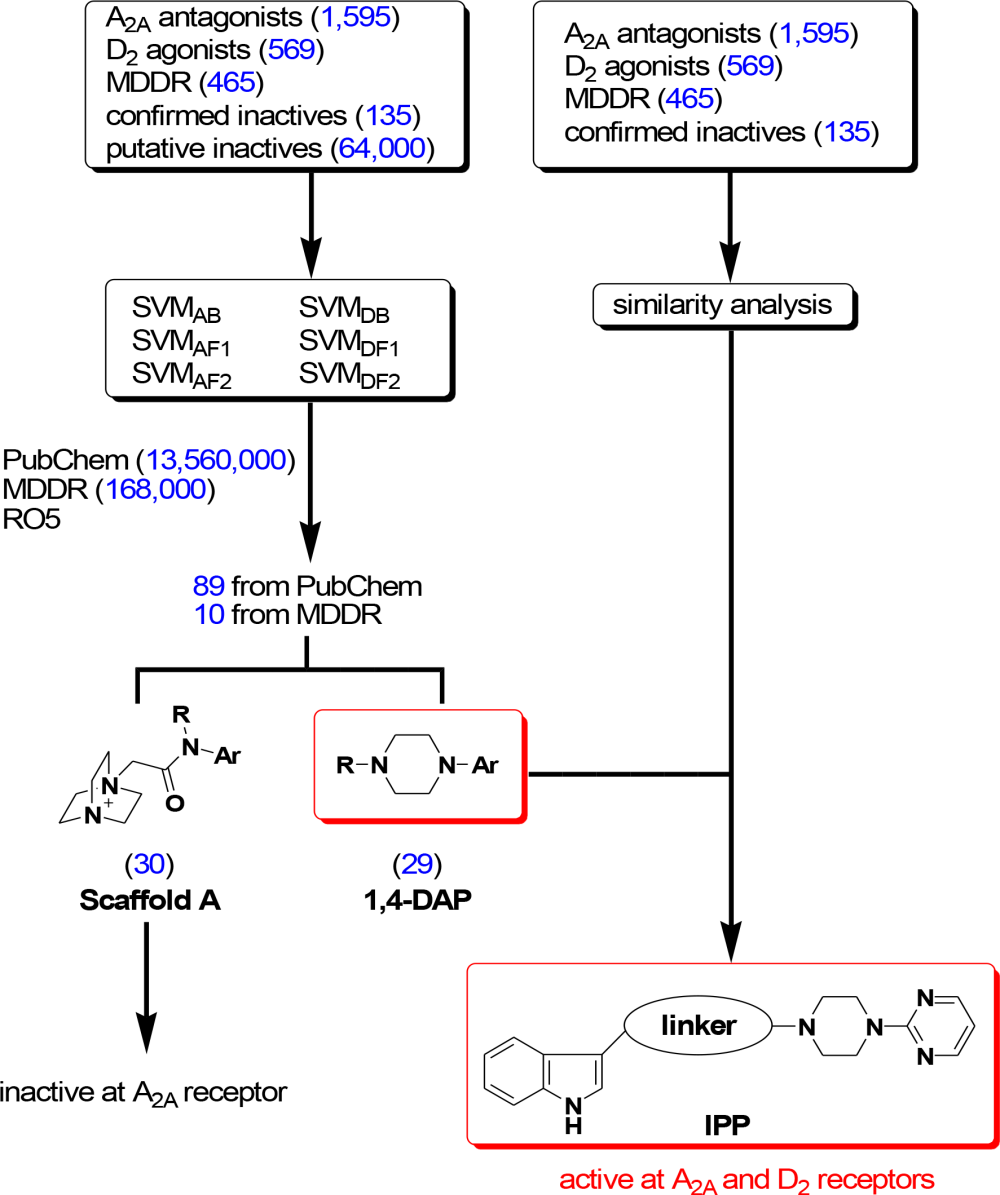
Flowchart of virtual screening. The data are also extrapolated from **S2–S8 Figs**.

With compound collection from literature and putative inactive families [20] at hand (135 in total, 96 from A_2A_ and 39 from D_2_ compounds), two ligand-based computational tools—SVM models and Tanimoto similarity-based clustering analysis—were used to analyze these collections. Six SVM models were developed and used for screening 13.56 million compounds in the PubChem database and 168,000 compounds in the MDDR database. A total of 172 hits (162 from PubChem, 10 from MDDR) were identified, and after filtering by Lipinski’s rule of 5, a total of 99 hits (89 from PubChem, 10 from MDDR) were collected (**S2 and S3 Tables**). Analysis of these 99 hits led to the identification that compounds bearing 1-aza-4-azoniabicyclo[2.2.2]octane moiety (Scaffold A, **Fig 2**) and compounds bearing 1,4-disubstituted aromatic piperazine (1,4-DAP, **Fig 2**) constituted the highest percentage in numbers—30.3% for Scaffold A and 29.3% for 1,4-DAP, respectively. Four representative compounds bearing Scaffold A were selected and tested at A_2A_ receptor through the *in vitro* radioligand displacement assay but did not show binding up to 100 μM. Therefore, the 1,4-DAP scaffold has become the focus of the present study.

In parallel, a Tanimoto similarity-based clustering analysis using two-dimensional fingerprints [21] was carried out on the collected A_2A_ antagonists and D_2_ agonists. A total of 1.5 million pairs of compounds were generated. Each pair is composed of an A_2A_ antagonist and a D_2_ agonist. The degree of structural similarity between an A_2A_ antagonist and a D_2_ agonist was calculated and expressed by Tanimoto coefficient (Tc). Based on their computed Tc values, the A_2A_ antagonists and D_2_ agonists were further clustered in a dendrogram to identify regions with high overlapping opportunities. Collectively, it was revealed that compounds having indole and pyrimidine placed at the two terminal ends with a linker of up to 4 carbon atoms (as suggested by the spacers used in both A_2A_ and D_2_ compounds collected in the original dataset [13–22]) had the potential to bind the two receptors simultaneously. These findings, together with the input of 1,4-DAP identified by SVM models, suggested that pyrimidine was the likely aromatic group in 1,4-DAP and the placement of an indole ring at the other terminal end may be important for binding at the two receptors. Hence, compounds with the substructure indole-piperazine-pyrimidine (IPP, **Fig 2**) were designed, synthesized, and evaluated in various *in vitro* and *in vivo* assays. Of note, small methyl groups were introduced at position 4 and 6 of the pyrimidine, as these substituents were reported to enhance the affinity profile at A_2A_ receptors by ~30 fold, while still displaying a very high structural similarity (Tc > 0.9) with a D_2_ ligand (**S1 Fig**).

### Chemistry

**Fig 3:**
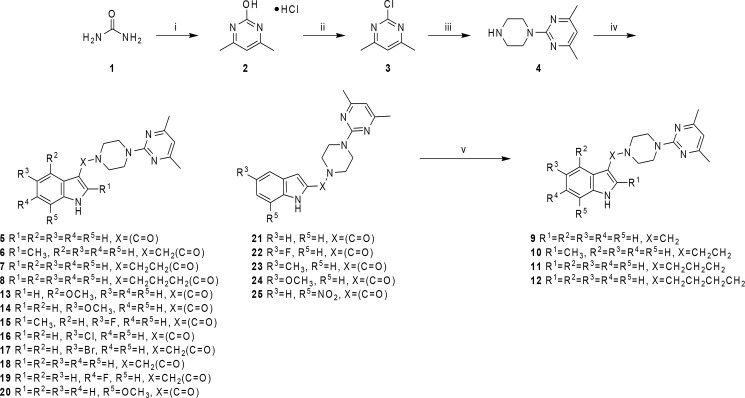
Reagents and conditions: (i) 2,4-pentanedione, 37% HCl, EtOH, reflux, 24 h, 78%; (ii) POCl_3_, reflux, 10 h, 91%; (iii) piperazine, K_2_CO_3_, H_2_O, 45–50°C, 4.5 h, 93%; (iv) indole-3-acid or indole-2-acid, EDC. HCl, EtOAc, DMF, 23 → 50°C, 4~4.5 h, 50~74%; (v) LiAlH_4_, THF, 0 → 23°C, 4–21.5 h, 76–97%.

The designed compounds with IPP scaffold were synthesized according to **Fig 3**. The mixture of urea **1** and 2,4-pentanedione under acidic conditions resulted in the formation of 4,6-dimethylpyrimidin-2-ol hydrochloride (**2**) with a 78% yield. Following this, treating **2** with phosphorus oxychloride gave 2-chloro-4,6-dimethylpyrimidine (**3**) with a 91% yield [23]. In order to minimize the formation of 2-[4-(4,6-dimethylpyrimidin-2-yl)piperazin-1-yl]-4,6-dimethylpyrimidine, five equivalents of piperazine were used for coupling with **3** under basic conditions. With 4,6-dimethyl-2-(piperazin-1-yl)pyrimidine (**4**) on hand, four indole-3-acids (i.e. indole-3-carboxylic acid, 2-methylindole-3-acetic acid, indole-3-propionic acid and indole-3-butyric acid) were selected for 1-ethyl-3-(3-dimethylaminopropyl)carbodiimide (EDC)-mediated amide formation to generate compounds **5–8** bearing different lengths of the linker between indole and piperazine rings. To study the effect of the carbonyl group on biological activities, reduction by lithium aluminium hydride (LiAlH_4_) was performed on **5–8** to give **9–12**. Similar procedures were adopted for the preparation of compounds **13–25**.

### Binding affinity studies at human adenosine receptors

**Fig 4:**
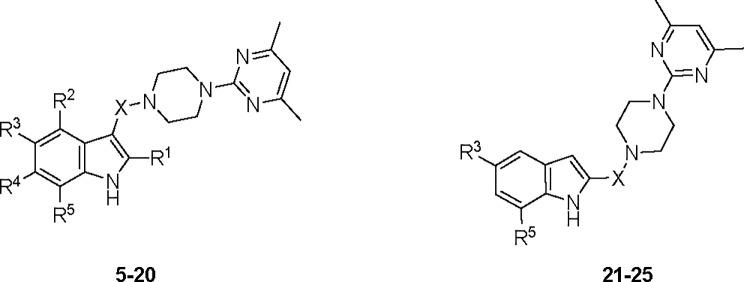
Structures of compounds 5–25 tested at human adenosine receptor subtypes.

The synthesized IPP compounds were tested in competition binding assays at human (h) A_1_, A_2A_, A_2B_ and A_3_ adenosine receptors expressed in Chinese Hamster Ovary (CHO) cells (**Fig 4**and **Table 1**). Based on the results obtained, it was shown that the presence of a carbonyl group between indole and piperazine rings has led to derivatives with higher affinity at the human A_2A_ (hA_2A_) receptor than the corresponding compounds without a carbonyl group (i.e. compound **5**, *K*_i_ hA_2A_ = 11.2 μM, hA_1_/hA_2A_ >9, hA_3_/hA_2A_ >9 *versus* compound **9**, *K*_i_ hA_2A_ > 30 μM; compound **6**, *K*_i_ hA_2A_ = 8.71 μM, hA_1_/hA_2A_ >11, hA_3_/hA_2A_ >11 *versus* compound **10**, *K*_i_ hA_2A_ = 34.4 μM, hA_1_/hA_2A_ >3, hA_3_/hA_2A_ = 1.16). This observation indicates the importance of the carbonyl group towards the binding affinity at the hA_2A_ receptor. In addition, it was found that extension of the length of the middle linker from two carbon atoms to three or four carbon atoms resulted in complete loss of A_2A_ affinity (compounds **7** and **8**: *K*_i_ hA_2A_ > 100 μM). Therefore, it was not unexpected that the reduced forms of compounds **7** and **8** (i.e. compounds **11** and **12**) did not show binding up to 100 μM.

**Table 1:** Binding affinity (*K*_i_, μM) of compounds 5–25 at human adenosine receptor subtypes.

Cpd	R^1^	R^2^	R^3^	R^4^	R^5^	X	hA_1_^*a*^	hA_2A_^*b*^	hA_2B_^*c*^	hA_3_^*d*^	hA_1_/hA_2A_	hA_3_/hA_2A_
**5**	H	H	H	H	H	C = O	>100	11. 20 (9.86–12.70)	>20	>100	>9	>9
**6**	CH_3_	H	H	H	H	CH_2_C = O	>100	8.71 (6.06–12.50)	>20	>100	>11	>11
**7**	H	H	H	H	H	CH_2_CH_2_C = O	N.D.	>100	>20	N.D.	N.D.	N.D.
**8**	H	H	H	H	H	CH_2_CH_2_CH_2_C = O	N.D.	>100	>20	N.D.	N.D.	N.D.
**9**	H	H	H	H	H	CH_2_	>100	>30	>20	>30	N.D.	N.D.
**10**	CH_3_	H	H	H	H	CH_2_CH_2_	>100	34.40 (23.80–49.90)	>20	39.80 (32.50–48.80)	>3	1.16
**11**	H	H	H	H	H	CH_2_CH_2_CH_2_	N.D.	>100	>20	N.D.	N.D.	N.D.
**12**	H	H	H	H	H	CH_2_CH_2_CH_2_CH_2_	N.D.	>100	>20	N.D.	N.D.	N.D.
**13**	H	OCH_3_	H	H	H	C = O	>100	18.80 (15.50–22.70)	>20	>30	>5	>2
**14**	H	H	OCH_3_	H	H	C = O	>100	12.70 (10.50–15.40)	>20	>30	>8	>2
**15**	CH_3_	H	F	H	H	CH_2_C = O	>100	18.20 (15.50–21.20)	>20	>30	>6	>2
**16**	H	H	Cl	H	H	C = O	>100	15.50 (13.50–17.80)	>20	19.50 (13.70–27.80)	>7	1.26
**17**	H	H	Br	H	H	CH_2_C = O	>100	>100	>20	>100	N.D.	N.D.
**18**	H	H	H	H	H	CH_2_C = O	>100	27.60 (21.40–35.70)	>20	>30	>4	>1
**19**	H	H	H	F	H	CH_2_C = O	>100	26.90 (20.30–35.60)	>20	>30	>4	>1
**20**	H	H	H	H	OCH_3_	C = O	>100	3.63 (2.01–6.57)	>20	>30	>28	>8
**21**	—	—	H	—	H	C = O	>100	>100	>20	>100	N.D.	N.D.
**22**	—	—	F	—	H	C = O	>100	>100	>20	>100	N.D.	N.D.
**23**	—	—	CH_3_	—	H	C = O	>100	>100	>20	>100	N.D.	N.D.
**24**	—	—	OCH_3_	—	H	C = O	>30	>30	>20	>30	N.D.	N.D.
**25**	—	—	H	—	NO_2_	C = O	4.82 (4.37–5.32)	29.70 (23.50–37.60)	>20	>30	0.16	>1

^*a*^Displacement of specific [^3^H]-2-chloro-6-cyclopentyladenosine ([^3^H]-CCPA) binding at human A_1_ receptors expressed in CHO cells (*n* = 3–6).

^*b*^Displacement of specific [^3^H]-5’-*N*-ethylcarboxamidoadenosine ([^3^H]-NECA) binding at human A_2A_ receptors expressed in CHO cells (*n* = 3–6).

^*c*^*K*_i_ values of the inhibition of NECA-stimulated adenylyl cyclase activity in CHO cells expressing human A_2B_ receptors (*n* = 3–6).

^*d*^Displacement of specific [^3^H]-2-hexyn-1-yl-*N*^*6*^-methyladenosine ([^3^H]-HEMADO) binding at human A_3_ receptors expressed in CHO cells (*n* = 3–6).

Data are expressed with 95% confidence limits. N.D., not determined.

Additionally, studies on substitution at the indole C4, C5, C6 and C7 positions were also conducted to investigate their effect on the hA_2A_ binding affinity by replacing the hydrogen atoms with substituent groups, including halogens and methoxy group. It was observed that such replacement did not significantly enhance the binding affinity at the hA_2A_ receptor as compared to that displayed by compounds **5** and **6**. For example, at the indole C4 position, introduction of a methoxy group (compound **13**, *K*_i_ hA_2A_ = 18.8 μM hA_1_/hA_2A_ >5, hA_3_/hA_2A_ >2) did not produce appreciable difference in hA_2A_ binding from corresponding derivative without the methoxy substitution (compound **5**, *K*_i_ hA_2A_ = 11.2 μM, hA_1_/hA_2A_ >9, hA_3_/hA_2A_ >9). At the indole C5 position, similar findings were noted with methoxy (compound **14**, *K*_i_ hA_2A_ = 12.7 μM, hA_1_/hA_2A_ >8, hA_3_/hA_2A_ >2 *versus* compound **5**), fluorine (compound **15**, *K*_i_ hA_2A_ = 18.2 μM, hA_1_/hA_2A_ >6, hA_3_/hA_2A_ >2 *versus* compound **6**), and chlorine (compound **16**, *K*_i_ hA_2A_ = 15.5 μM, hA_1_/hA_2A_ >7, hA_3_/hA_2A_ = 1.26 *versus* compound **5**) substitutions. However, the presence of bromine at the C5 position was found to be detrimental to the hA_2A_ binding affinity (compound **17**, *K*_i_ hA_2A_ > 100 μM *versus* compound **18**, *K*_i_ hA_2A_ = 27.6 μM, hA_1_/hA_2A_ >4, hA_3_/hA_2A_ >1). It is probable that the large size of bromine at the C5 position causes steric clash with adjacent residues in the binding pocket, thus leading to ineffective binding.

Likewise, at the indole C6 position, introduction of fluorine did not demonstrate significant change in the hA_2A_ affinity (compound **19**, *K*_i_ hA_2A_ = 26.9 μM, hA_1_/hA_2A_ >4, hA_3_/hA_2A_ >1 *versus* compound **18**). Nonetheless, it was found that methoxy group substitution at the indole C7 position had led to a 3-fold improvement in hA_2A_ affinity (compound **20**, *K*_i_ hA_2A_ = 3.63 μM, hA_1_/hA_2A_ >28, hA_3_/hA_2A_ >8 *versus* compound **5**). The enhanced binding observed in compound **20** could be attributed to the oxygen atom of the methoxy substituent participating in hydrogen bonds with neighbouring residues in the binding cavity.

Furthermore, the binding affinity of compound **6** was compared with that of compound **18** (compound **6**, *K*_i_ hA_2A_ = 8.71 μM hA_1_/hA_2A_ >11, hA_3_/hA_2A_ >11 *versus* compound **18**, *K*_i_ hA_2A_ = 27.6 μM, hA_1_/hA_2A_ >4, hA_3_/hA_2A_ >1). From such comparison, it was noted that the absence of a methyl group at the indole C2 position in compound **18** has led to a 3.2-fold decrease in hA_2A_ affinity. This finding suggests that the C2-methyl group contributes to the binding affinity at the hA_2A_ receptor to a certain extent.

An additional study was also carried out to determine the effect of a linker at the indole C2 position towards the hA_2A_ binding affinity. This provides better understanding of differences in indole C2 and C3 extension towards affinity at the hA_2A_ receptor. From the results obtained, it was revealed that except for compound **25**, the incorporation of linker at the indole C2 position rendered derivatives (compounds **21–24**) inactive at the hA_2A_ receptor. This observation suggested the piperazine-pyrimidine moiety was not well tolerated when it was extended from the indole C2 position. Notably, 7-nitro indolyl derivative **25** showed modest affinity at the hA_2A_ receptor (*K*_i_ hA_2A_ = 29.7 μM, hA_1_/hA_2A_ = 0.16, hA_3_/hA_2A_ >1). It is speculated that the oxygen atoms on the nitro group could likely form hydrogen bonding with adjacent water molecules or neighbouring residues in the binding pocket. In fact, such 7-nitroindole-2-susbtituted derivative (compound **25**, with the highest hA_2A_ affinity in the indole-2 series) is reminiscent of 7-methoxyindole-3-substituted derivative (compound **20**, with the highest hA_2A_ affinity in the indole-3 series). The oxygen atoms of methoxy and nitro group could possibly engage in similar hydrogen bonding interaction with residues in the vicinity.

### Binding affinity studies at human dopamine D_2_ receptor

**Fig 5:**
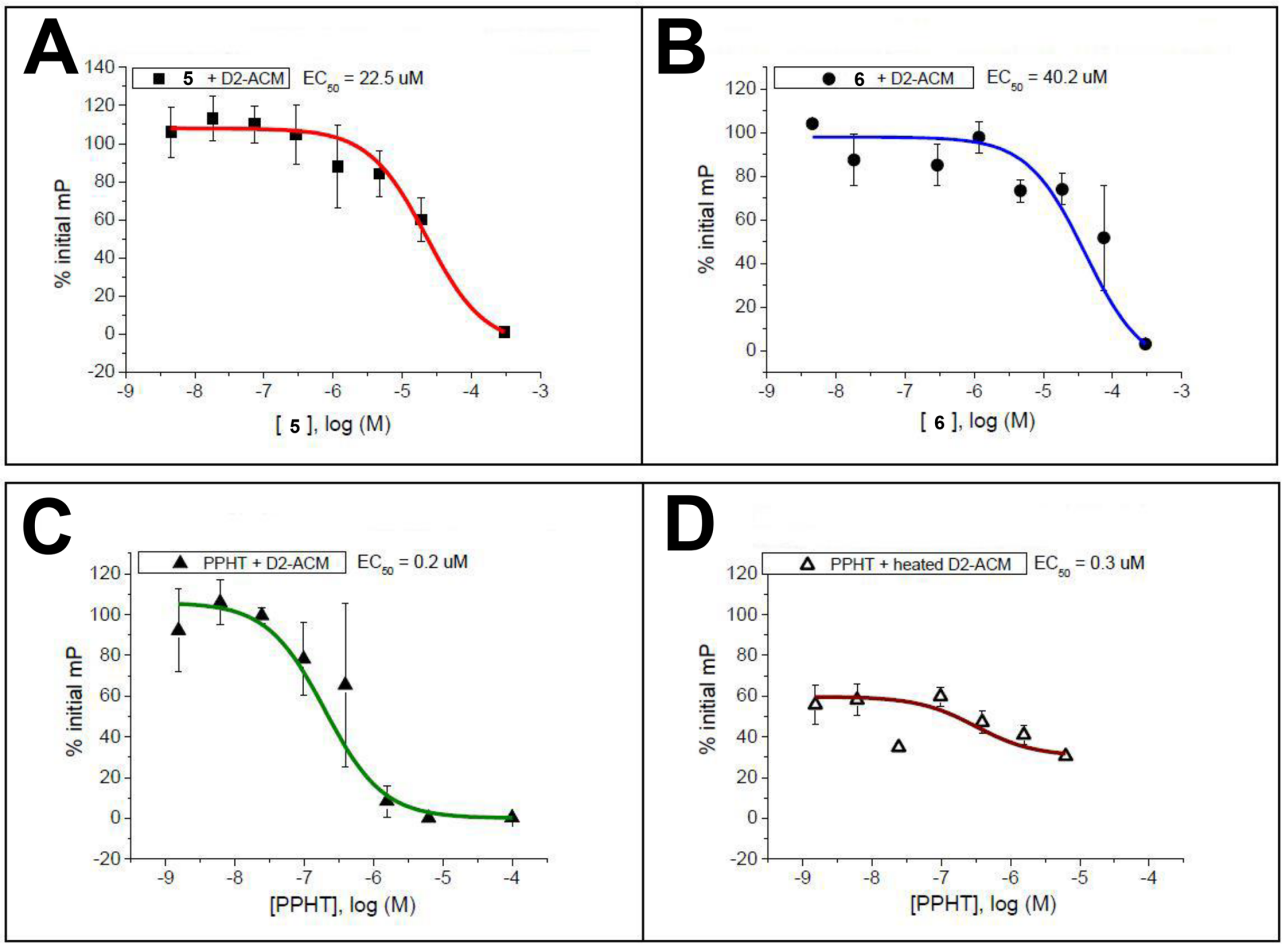
Dose-response curves of compound **5** (A), compound **6** (B) and (±)-PPHT.HCl (C) in D_2_R-proteopolymersomes-based fluorescence polarisation (FP) competition assay with BODIPY-NAPS. The non-binding control (D) was achieved by denaturing the D_2_R-proteopolymersomes with heat, resulting in a curve that fluorescence intensity did not decrease much when the concentration of (±)-PPHT.HCl increased. For the highest concentrations, experiments were repeated only in duplicate due to solubility issues.

In addition to the human A_2A_ receptor binding affinity assay, the newly synthesized IPP derivatives were further examined for their binding affinity toward human dopamine D_2_ receptor. Among these, compounds **5** and **6** with hA_2A_ binding affinity in the low micromolar range were selected for the polymersome-based dopamine D_2_ receptor binding assay. In such assay, boron-dipyrromethene *N*-(*p*-aminophenethyl)spiperone (BODIPY-NAPS, a fluorescent ligand) was incubated with D_2_ receptor-functionalised polymersomes; spiperone is a selective D_2_-like antagonist with reported *K*_i_ of 0.06 nM for D_2_ receptor [24]. The mixture was then incubated with solutions containing eight different concentrations (ranging from 3 nM to 0.3 mM) of compounds **5** and **6**, and the measured fluorescence intensity against ligand concentration was plotted accordingly (**Fig 5A and 5B**). Notably, dose-dependent reduction in fluorescence was observed for both compounds, indicating that strong binding (*K*_i_ = 0.06 nM) of spiperone with D_2_ receptor can be competitively replaced by compounds **5** and **6**.

Furthermore, (±)-2-(*N*-phenethyl-*N*-propyl)amino-5-hydroxytetralin hydrochloride ((±)-PPHT.HCl), a potent dopamine D_2_ receptor agonist with a *K*_i_ of 13.3 nM determined by competition binding experiments with [^3^H]spiperone [24], was tested for its ability to displace BODIPY-NAPS. Similarly, a sigmoidal decrease in fluorescence with increasing concentration of (±)-PPHT.HCl was noted (**Fig 5C**); this observation was characteristic of D_2_ agonist interactions in the D_2_R-functionalized polymersomes. As illustrated in **Fig 5A–5C**, the dose-response curves of compounds **5** and **6** coherently resembled that of the (±)-PPHT.HCl. The EC_50_ values of compound 5, compound 6 and (±)-PPHT.HCl were determined to be 22.5 μM, 40.2 μM and 0.2 μM, respectively. Besides, treatment of denatured D_2_R-proteopolymersomes with PPHT.HCl was performed (**Fig 5D**) for comparison with **Fig 5C**, showing the necessity of proteopolymersomes’ integrity.

### Adenylyl cyclase inhibition studies at human dopamine D_2_ receptors

D_2_ receptor activation is mediated by heterotrimeric (i.e. α, β and γ subunits) G_i/o_ proteins. In the absence of endogenous dopamine or agonists, G_α_ is bound to guanosine 5’-diphosphate (GDP) and G_βγ_. Upon D_2_ receptor activation, the conformational change of the receptor results in the GDP release, guanosine 5’-triphosphate (GTP) binding, and dissociation of G_α_ from G_βγ_. The released G_α_ then interacts with and inhibits adenylyl cyclase, leading to a decrease in the adenosine 3’,5’-cyclic monophosphate (cAMP) production. Thus, measuring the extent to which a compound inhibits cAMP accumulation has been one of the functional assays for D_2_ receptor activation [25].

**Fig 6:**
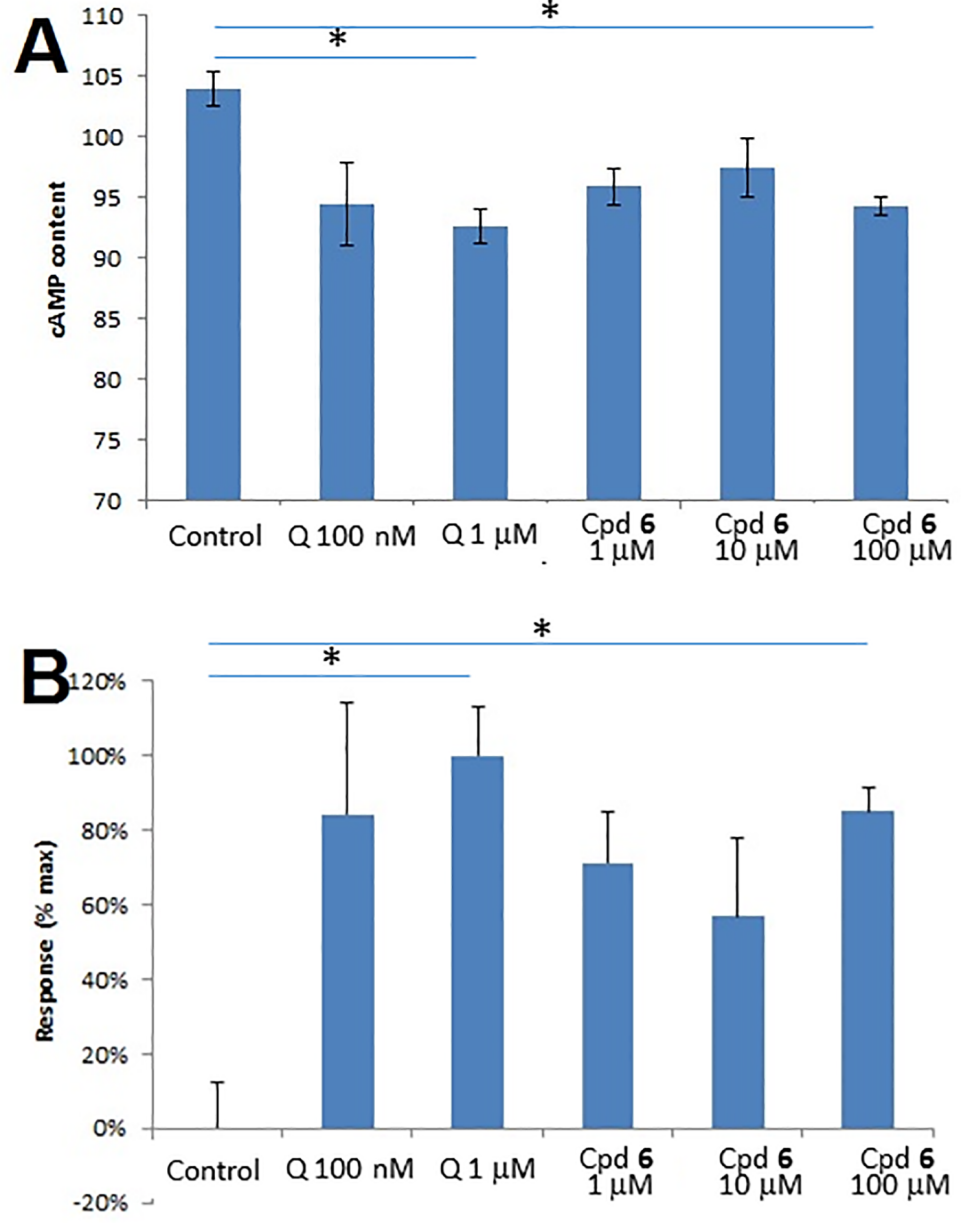
Inhibition of cAMP accumulation induced by compound 6 in comparison with the reference compound quinpirole (Q). The CHO cells were transfected with D_2_ receptor, pre-treated with the indicated concentrations of quinpirole or compound **6**, and activated with forskolin/IBMX. (A) The cAMP concentration (in the unit of picomole) measured by ELISA. (B) Normalisation to control (0%) and 1 μM quinpirole (100%). *p<0.05. Error bars = standard error of the mean (SEM).

Hence, adenylyl cyclase inhibition studies on compound **6** were carried out to determine whether the compound was able to activate D_2_ receptors. In this functional assay, Chinese Hamster Ovary (CHO) cells were transfected with D_2_ receptor, pre-treated with compound **6** or quinpirole, and stimulated with forskolin and 3-isobutyl-1-methylxanthine (IBMX). Quinpirole, a potent D_2_ receptor agonist with a *K*_i_ of 4.8 nM [12], acted as the reference agent. Forskolin (an activator of adenylyl cyclase) and IBMX (an inhibitor of phosphodiesterase) were used to elevate the basal levels of cAMP. As shown in **Fig 6A**, the control referred to the high cAMP content resulted from the stimulation with forskolin/IBMX in the absence of either quinpirole or compound **6**. Subsequently, different concentrations of either quinpirole or compound **6** were added to investigate whether, and if so, to which extent the added ligand could reverse the cAMP effect induced by forskolin/IBMX. The concentration-dependent decrease in cAMP accumulation when the D_2_ receptor-expressing cells were treated with quinpirole suggested that quinpirole-induced D_2_ receptor conformation caused the G_i_ protein to bind and inhibit the adenylyl cyclase [26]. Activation with quinpirole at the concentration of 1 μM resulted in an 11% decrease (calculated by (103.97–92.63)/103.97 = 11%) in intracellular cAMP concentration with p-value of 0.0232, and the result of 1 μM quinpirole was normalised relative to the control (**Fig 6B**). We evaluated the relative efficacy of our compounds by normalizing the resulting cAMP levels relative to that of forskolin/IBMX-treated cells (0%) and 1 μM quinpirole-mediated inhibitory response (100%) (**Fig 6B**). Three concentrations of compound **6** (1 μM, 10 μM and 100 μM) were evaluated, and their respective capability to inhibit cAMP accumulation was measured. There was no concentration-dependent response for compound **6** observed in this cAMP assay. The optimal inhibition occurred at 100 μM of compound **6** (p = 0.0232), which showed comparable potency to 100 nM of quinpirole. In **Fig 6B**, the y-axis values for all three concentrations of compound **6** were above zero, suggesting that compound **6** inhibited cAMP accumulation induced by forskolin/IBMX and therefore acted as a D_2_ receptor agonist.

### *Drosophila* models of PD

Although most PD cases occur in a sporadic manner, there are increasing studies indicating the association of PD with genetic etiologies [27]. In particular, mutations in both *Parkin* and *LRRK2* genes have been recognized to be the predominant causes for early- [28] and late-onset [29] hereditary PD cases, respectively. Of all the genetic models developed for investigating the molecular events regulated by PD mutant genes, the *Drosophila* model has gained significant traction due to its highly conserved homologues of many human genes. In fact, both *Parkin* [30] and *LRRK2* [31] mutants in *Drosophila* models faithfully phenocopy many of the characteristics of PD, including reduction of dopaminergic (DA) neurons in the brain. Moreover, the closest human homologue of the *Drosophila* adenosine receptor, DmAdoR, is hA_2A_ receptor, and hence using *Drosophila* models to assess A_2A_ ligands such as compounds **5** and **6** provides better correlation. Furthermore, D_2_-like receptors [32] have been shown to regulate locomotion in the *Drosophila* [33]. In view of the relevance of *Drosophila* to human A_2A_ and D_2_ receptors, this *in vivo* system was used to test the efficacy of compounds **5** and **6**.

**Fig 7:**
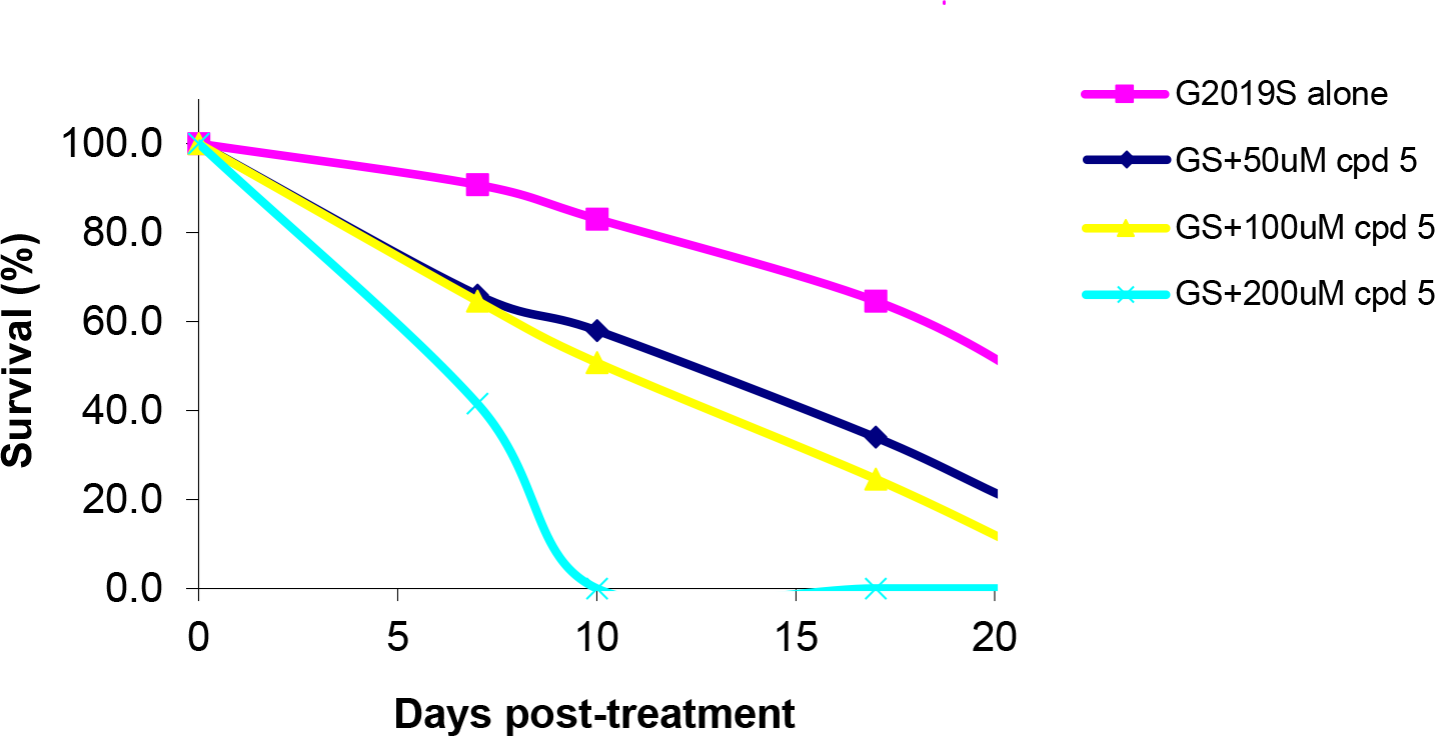
Survival percentages of mutant LRRK2 G2019S flies after 7 days, 10 days and 17 days of treatment with various concentrations (50 μM, 100 μM, 200 μM) of compound 5.

Notably, there is high prevalence of G2019S mutant in the LRRK2-associated PD cases [34,35]. Studies have shown that expression of the LRRK2-G2019S mutantion in *Drosophila* caused more severe PD phenotypes, including locomotor dysfunction, loss of dopaminergic neurons, and early mortality, relative to that of wild-type LRRK2 [31]. Prior to evaluating compounds **5** and **6** in the *Drosophila* model of PD, the effect of compound concentrations on fly viability was studied. At the ligand concentration of 500 μM, both compounds were lethal to G2019S flies (data not shown). Lower ligand concentrations were attempted, and at concentrations in the 50–100 μM range, whilst treatment with compound **6** still resulted in toxicity, compound **5** was able to delay the G2019S fly mortality (**Fig 7**). As there was no remarkable difference in the survival rate between 50 and 100 μM for compound **5**, this range was selected for compound **5** for the subsequent climbing assay and quantification of DA neurons.

**Fig 8:**
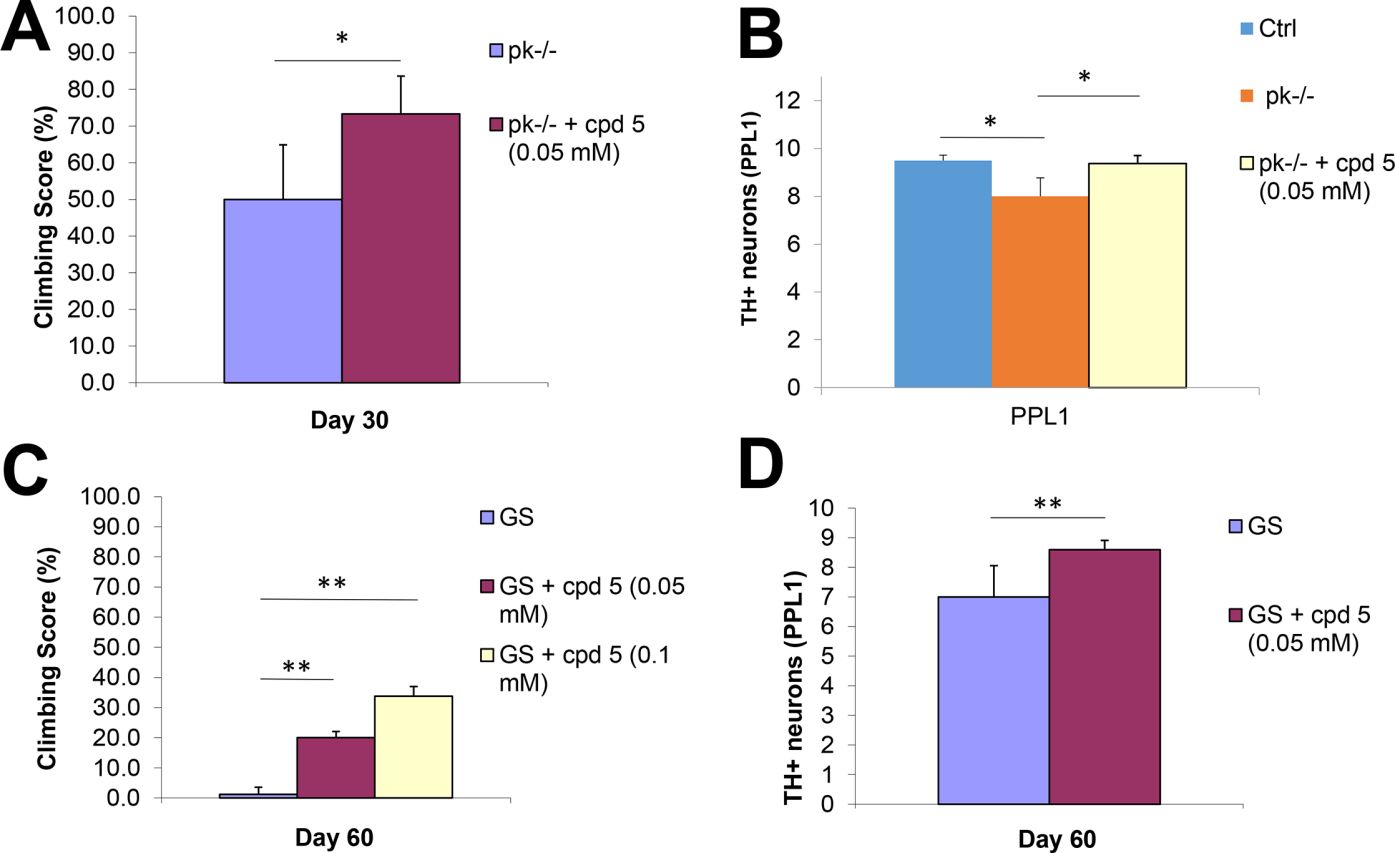
Treatment of compounds **5** in both parkin-null (A and B) and mutant LRRK2 G2019S-expressing (C and D) flies. A: Climbing score of untreated and compound **5** (50 μM)-treated parkin-null flies (where 100% is the climbing score of normal flies). B: DA neuronal count (PPL1 cluster) of normal flies (control), parkin-null flies, and compound **5** (50 μM)-treated parkin-null flies. C: As in A, except that parkin-null flies were substituted with LRRK2 G2019S-expressing flies (the control is represented by the mutant flies). Two concentrations, 50 μM and 100 μM, of compound **5** were tested. D: As in B, except that parkin-null flies were substituted with LRRK2 G2019S-expressing flies. (*p < 0.05, **p < 0.01).

Parkin-null (pk-/-) flies were first used to evaluate compound **5**. It was found that compound **5**-treated parkin-null flies not only exhibited improvement in climbing scores compared with untreated mutant flies (**Fig 8A**), but they also showed reduction of DA neuron loss in the PPL1 cluster [36] (**Fig 8B**), the cluster used widely in the parkin-null flies. Compound **5** was subsequently examined for its ability to ameliorate LRRK2 G2019S-induced PD phenotypes. It was noted that compound **5**-treated LRRK2 G2019S flies displayed improvement in climbing (in a concentration-dependent manner) (**Fig 8C**) as well as mitigation of DA neuron degeneration (**Fig 8D**). Taken together, these findings indicate that compound **5** acts as a suppressor of DA neuron dysfunction in two *Drosophila* genetic PD models—one with parkin-null which represents recessive PD, and one with transgene LRRK2 G2019S-mutant, which represents dominant PD.

Notwithstanding the promising results that we have obtained from the *Drosophila* system, we recognized that there are inherent limitations of the fly model. For example, by virtue of their vastly different brain architecture from their human counterparts, fly PD models cannot recapitulate fully the phenotypic and pathologic features of the human condition. Moreover, some known disease-associated factors like α-synuclein are not expressed in the fly brain. Nevertheless, they do exhibit salient features of PD such as age-dependent dopaminergic neurodegeneration and associated locomotion deficits. Importantly, like PD patients, fly PD models also respond positively to the L-DOPA treatment, making them suitable model for rapid drug evaluation.

### Toxicity studies

In the toxicity studies, *in vitro* assays for mutagenicity and cytotoxicity were performed on compounds **5** and **6**. These compounds were investigated for their mutagenic potential through the Ames assay [37]. Two *Salmonella* strains TA98 and TA100 with pre-existing mutations (His^-^) were selected. Both strains are unable to synthesize histidine (one of the growth requirements for these strains) and therefore cannot form colonies on the agar plate. If the incubation of one such strain with a given chemical results in the formation of many colonies, it would suggest that chemical induces back-mutation (His^-^ → His^+^) and is therefore considered as a mutagen.

Together with the compounds, the *Salmonella* strains were incubated in the presence (+S9) and absence (–S9) of a metabolizing system “S9 mix” consisting of 9000 *g* supernatant fraction of rat liver microsomes, nicotinamide adenine dinucleotide phosphate (NADP) and cofactors. Exogenous “S9 mix” was included due to the lack of CYP metabolizing enzymes in *Salmonella typhimurium*. In the assay, agar plates +S9 and–S9 were used to detect pro-mutagens for cases where the native chemical was not mutagenic but its metabolites were mutagens, and direct-acting mutagens, respectively. 2-aminoanthracene (2-AA) was used as positive control chemical for TA98 +S9 (**S9B Fig**) and TA100 +S9 (**S9D Fig**), while 4-nitroquinoline *N*-oxide (4-NQO) was included as positive control chemical for TA98 –S9 (**S9A Fig**) and TA100 –S9 (**S9C Fig**). Methotrexate was used as the negative control for all four incubations (**S9A–S9D Fig**), and DMSO was used as the solvent control (vehicle). As shown in **S9 Fig**, the number of His^+^ revertants induced by compounds **5** and **6** at two concentrations, 10 μM and 100 μM, was even lower than that induced by methotrexate in each incubation, suggesting that these two compounds have no significant mutagenicity and are therefore considered as non-mutagens.

Besides the mutagenicity study, compounds **5** and **6** were also subjected to cell viability assays using transforming growth factor-alpha mouse hepatocyte (TAMH) and HL-1 cardiomyocyte to assess the cytotoxic potential. TAMH lines were treated with compounds **5** and **6** in eight different concentrations (100 μM, 33.3 μM, 11.1 μM, 3.7 μM, 1.23 μM, 0.41 μM, 0.13 μM, 0.045 μM), and the percentage of viable cells was plotted against the logarithm of ligand concentration (**S10B and S10C Fig**). Both compounds at concentrations less than 30 μM were shown to be non-cytotoxic in the TAMH lines. Acetaminophen, a drug with direct hepatotoxic potential [38], was used as the positive control in this assay (**S10A Fig**). Similar to the viability study using TAMH lines, there was no toxicity observed at ligand concentrations less than 30 μM in the HL-1 cell (**S10E and S10F Fig**). Doxorubicin, a chemotherapeutic agent known to cause dose-dependent cardiotoxicity [39], was used as the positive control in this assay (**S10D Fig**).

### Aqueous solubility studies

**Fig 9:**
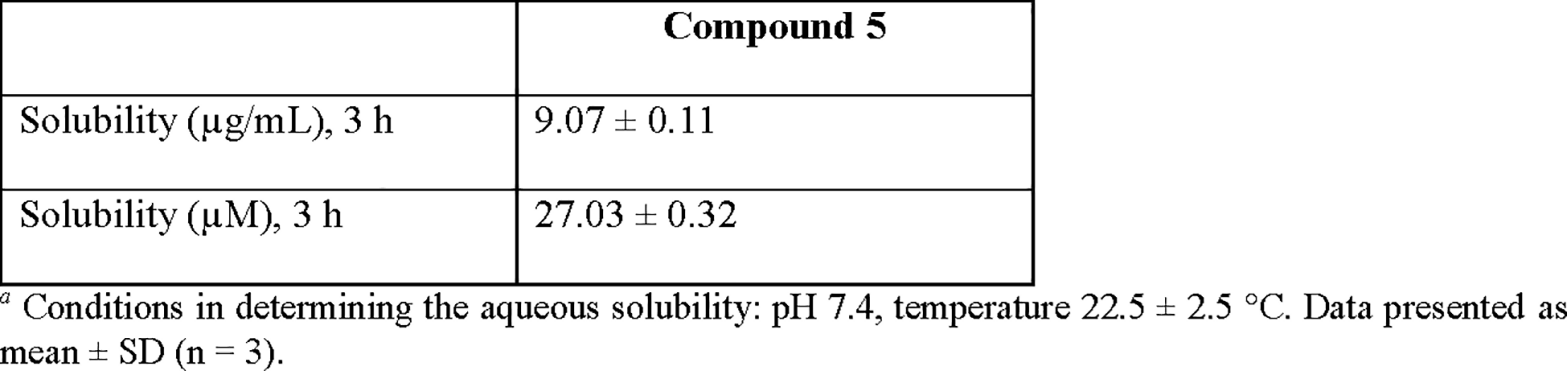
The aqueous solubility of compound 5^*a*^.

In addition to the pharmacological assays, aqueous solubility of compound **5** was determined and the results were presented in **Fig 9**. The solubility of compound **5** was found to be 9.07 ± 0.11 μg/mL (27.03 ± 0.32 μM) at 3-hour interval. Based on the solubility guideline for oral absorption by Kerns *et al*. [40], the following solubility ranges are suggested for lead compound at the drug discovery stage: < 10 μg/mL (low solubility), 10~60 μg/mL (moderate solubility), > 60μg/mL (high solubility). As such, compound **5** is therefore deduced to exhibit marginally low aqueous solubility at ambient temperature in pH 7.4. Nonetheless, the structural modification on compound **5** is underway in our laboratory to enhance its aqueous solubility while improving its binding profiles at both adenosine A_2A_ adenosine receptors and dopamine D_2_ receptors.

## Conclusion

The significant role of human adenosine A_2A_ receptor and dopamine D_2_ receptor in the pathogenesis of PD, together with emerging paradigm of drug actions on multiple receptors has spurred the discovery of new compounds to modulate both G protein-coupled receptors. In our study, a new series of indole-piperazine-pyrimidine (IPP) derivatives targeting the two receptors has been successfully synthesized and evaluated through integration of computational tools, synthetic chemistry and pharmacological assays. Compounds **5** and **6** have demonstrated affinity at the human adenosine A_2A_ receptor in the low micromolar range (*K*_i_ hA_2A_ = 8.7–11.2 μM). Based on the structure-affinity relationship studies as illustrated above, three important structural features of IPP-containing compounds for the hA_2A_ binding are derived:

1. At the indole C2 position, the presence of methyl group confers better hA_2A_ binding affinity than that of hydrogen.
2. The linker bridging indole C3 and piperazine nitrogen should contain a carbonyl group, and the length of the linker is limited to one or two carbon atoms.
3. At the indole C7 position, methoxy group improves the hA_2A_ binding affinity.

In the proteopolymersome-based D_2_ receptor binding assay, compounds **5** and **6** have displayed binding to the dopamine D_2_ receptor with EC_50_ of 22.5–40.2 μM. In addition, a functional assay for D_2_ receptor activation was conducted with compound **6** which demonstrated that inhibition of cAMP accumulation in CHO cells by 100 μM was comparable to the activity induced by 100 nM of quinpirole. Such observation has highlighted the D_2_ receptor agonistic activity of compound **6**. Further *in vivo* testing of compound **5** in the *Drosophila* model of PD at 50 μM showed improvement in movement as well as mitigation of the loss of dopaminergic neurons. In the *in vitro* toxicity studies, compounds **5** and **6** did not exhibit mutagenicity up to 100 μM, nor hepatotoxicity or cardiotoxicity up to 30 μM.

In summary, our study has led to the identification of novel IPP scaffolds acting on both human adenosine A_2A_ and dopamine D_2_ receptors. Structural optimization of this novel scaffold is deemed beneficial in providing insights into structural requirements for future development of new anti-parkinsonian agents.

## Experimental section

### Chemistry

#### General

Reactions were constantly monitored by thin layer chromatography (TLC) on silica gel (precoated 60 F254 Merck plates) and carried out under a nitrogen atmosphere. All chemicals are commercial products from Sigma Aldrich or Alfa Aesar. Column chromatography was performed using silica gel 60 (Merck, 70–230 mesh). Compounds were dissolved in HPLC-grade MeOH for accurate mass analysis using ESI time-of-flight (TOF) ionisation mode. ^1^H and ^13^C NMR spectra were determined in deuterated chloroform (CDCl_3_) or deuterated dimethylsulfoxide (DMSO-*d*_6_) using Bruker DPX Ultrashield NMR (400 MHz) spectrometer, with chemical shifts given in parts per million (δ) downfield relative to the central peak of the solvents, and *J* values (coupling constants) were given in Hz. The following abbreviations were used: s = singlet, d = doublet, t = triplet, m = multiplet, br = broad, td = triplet of doublets. High-performance liquid chromatography (HPLC) analysis was carried out for compounds used in biological assays. For HPLC (1): Hewlett-Packard series 1050 HPLC system equipped with a HP-1050 quaternary pump, a degasser, diode array detector, a HP-1100 autosampler, and a LiChrosorb reversed phase C18 (5 μm) column (4.6 × 250 mm) with solvents being CH_3_CN/H_2_O. For HPLC (2) and HPLC (3): Agilent HPLC 1200 series instrument on a Zorbax SB-C18, 4.6 mm × 250 mm, 5 μm column with solvents being CH_3_CN/H_2_O (0.1% v/v CF_3_COOH) and MeOH/H_2_O (0.1% v/v CF_3_COOH) for HPLC (2) and HPLC (3), respectively. All HPLC samples were prepared by dissolving them in HPLC-grade MeOH. The analysis was performed at 30°C, and the ultraviolet detection was made at wavelength 254 nm. The separations were carried out using gradient elution. The HPLC methods are as follows. HPLC (1): injection volume 5 μL, stop time 20 min, flow rate 1 mL/min, a gradient of 35 → 100% CH_3_CN for the 0–17 min period and back to CH_3_CN/H_2_O (3:7) at 20 min. HPLC (2): injection volume 20 μL, stop time 15 min, flow rate 0.5 mL/min, a gradient of 5 → 95% CH_3_CN for the 0–12 min period and back to CH_3_CN/H_2_O (5:95) at 15 min. HPLC (3): injection volume 20 μL, stop time 15 min, flow rate 0.5 mL/min, a gradient of 5 → 95% MeOH for the 0–12 min period and back to MeOH/H_2_O (5:95) at 15 min. The purities of all tested compounds were > 95% measured by the peak area of the product divided by that of the total peak areas.

#### 4,6‐Dimethylpyrimidin‐2‐ol hydrochloride (2)

To a suspension of urea **1** (1000 mg, 16.65 mmol) in EtOH (10 mL) were added 2,4-pentanedione (1885 μL, 18.32 mmol) and concentrated hydrochloric acid (2775 μL, 33.3 mmol), and the resulting clear, colourless solution was stirred and refluxed under N_2_ for 24 h. The mixture was cooled to room temperature and filtered. The filter cake was washed with EtOH and Et_2_O, and dried in the vacuum oven to afford compound **2** (2096 mg, 78% yield) as an off-white crystalline solid. ^1^H NMR (400 MHz, DMSO-*d*_6_) δ 6.72 (s, 1H), 2.47 (s, 6H).

#### 2‐Chloro‐4,6‐dimethylpyrimidine (3)

The mixture of compound **2** (1998 mg, 12.44 mmol) and phosphorus oxychloride (20 mL) was refluxed for 10 h. The remaining phosphorus oxychloride was evaporated to get a brown oil in the flask. The mixture was cooled in an ice bath, and concentrated aqueous KOH solution was added dropwise cautiously with stirring, until the litmus paper showed pH value that is approximately 8. Diethyl ether (30 mL) was added, and the mixture was stirred for 2.5 h. The water layer was extracted with Et_2_O (3 × 30 mL) and EtOAc (3 × 30 mL). The organic layers were combined, washed with brine, dried over Na_2_SO_4_, filtered and concentrated under reduced pressure. The resulting yellow liquid was put in an ice bath under vacuum to afford compound **3** (1606 mg, 91% yield) as yellow crystals. ^1^H NMR (400 MHz, DMSO-*d*_6_) δ 7.33 (s, 1H), 2.42 (s, 6H).

#### 4,6‐Dimethyl‐2‐(piperazin‐1‐yl)pyrimidine (4)

Into a 250 mL round-bottom flask were charged K_2_CO_3_ (3849 mg, 27.85 mmol), piperazine (10903 mg, 126.58 mmol), and H_2_O (180 mL). The mixture was heated at 45–50°C until it became a clear solution. Compound **3** (3610 mg, 25.32 mmol) was divided into four portions, and each portion was added into the mixture in one-hour interval. After the addition of all amounts of compound **3**, the reaction mixture was cooled to room temperature and stirred overnight. The white precipitate was filtered, and the filtrate was collected. The filtrate underwent solid/liquid extraction with EtOAc (3 × 225 mL). Organic layers were collected, washed with brine, dried over Na_2_SO_4_, filtered, and concentrated under reduced pressure to give compound **4** (4536 mg, 93% yield) as a white crystalline solid. ^1^H NMR (400 MHz, DMSO-*d*_6_) δ 6.36 (s, 1H), 3.62 (t, *J* = 5.2 Hz, 4H), 2.69 (t, *J* = 5.2 Hz, 4H), 2.20 (s, 6H).

#### General procedure for the coupling reactions to obtain compounds 5–8 and 13–25

To a suspension of indole acid and EDC.HCl were added EtOAc and two drops of DMF, and the mixture was stirred for 1 h at 23°C followed by the addition of compound **4**. The reaction was heated at 50°C for 3 h, and solvents were evaporated to dryness under high vacuum. Water was added, sonicated, and filtered. The filter cake was washed with hexane/EtOAc and further purified by crystallization from MeOH/EtOH/Et_2_O/EtOAc.

#### 3‐[4‐(4,6‐Dimethylpyrimidin‐2‐yl)piperazine‐1‐carbonyl]‐1*H*‐indole (5)

Yield: 360 mg (50%) as white crystal (re-crystallization from MeOH/Et_2_O/EtOAc). ^1^H NMR (400 MHz, DMSO-*d*_6_) δ 11.61 (br s, 1H), 7.74–7.72 (m, 2H), 7.45 (d, *J* = 8 Hz, 1H), 7.16 (td, *J* = 7.2, 1.2 Hz, 1H), 7.10 (td, *J* = 7.2, 1.2 Hz, 1H), 6.44 (s, 1H), 3.82–3.80 (m, 4H), 3.71–3.69 (m, 4H), 2.24 (s, 6H). ^13^C NMR (100 MHz, DMSO-*d*_6_) δ 166.8 (2 × C), 165.7, 161.1, 135.7, 128.1, 126.1, 121.9, 120.3, 120.2, 111.9, 109.6, 109.1, 43.5 (2 × CH_2_), 40.1–38.9 (2 × CH_2_), 23.7 (2 × CH_3_). HRMS-ESI (*m*/*z*) [M + H]^+^ calcd for C_19_H_22_N_5_O, 336.1819; found, 336.1824 (Δ = -1.6 ppm). [M + Na]^+^ calcd for C_19_H_21_N_5_NaO, 358.1638; found, 358.1639 (Δ = -0.2 ppm). HPLC (1): t = 5.6 min, 96.7% purity. HPLC (3): t = 15.5 min, 98.3% purity.

#### 1‐[4‐(4,6‐Dimethylpyrimidin‐2‐yl)piperazin‐1‐yl]‐2‐(2‐methyl‐1*H*‐indol‐3‐yl)ethan‐1‐one (6)

Yield: 1130 mg (58%) as light brown crystal (re-crystallization from MeOH/EtOAc (3:1)). ^1^H NMR (400 MHz, DMSO-*d*_6_) δ 10.80 (br s, 1H), 7.45 (d, *J* = 7.6 Hz, 1H), 7.22 (d, *J* = 7.6 Hz, 1H), 6.97 (td, *J* = 7.2, 1.2 Hz, 1H), 6.90 (td, *J* = 7.2, 1.2 Hz, 1H), 6.41 (s, 1H), 3.74 (s, 2H), 3.62–3.61 (m, 2H), 3.52–3.49 (m, 6H), 2.34 (s, 3H), 2.20 (s, 6H). ^13^C NMR (100 MHz, DMSO-*d*_6_) δ 169.6, 166.8 (2 × C), 160.8, 135.1, 132.6, 128.2, 120.0, 118.2, 117.8, 110.3, 109.1, 104.0, 45.2, 43.3, 43.2, 41.2, 30.0, 23.6 (2 × CH_3_), 11.5. HRMS-ESI (*m*/*z*) [M + H]^+^ calcd for C_21_H_26_N_5_O, 364.2132; found, 364.2123 (Δ = 2.4 ppm). [M + Na]^+^ calcd for C_21_H_25_N_5_NaO, 386.1951; found, 386.1940 (Δ = 3.0 ppm). HPLC (1): t = 9.8 min, 99.9% purity. HPLC (3): t = 15.6 min, 99.6% purity.

#### 1‐[4‐(4,6‐Dimethylpyrimidin‐2‐yl)piperazin‐1‐yl]‐3‐(1*H*‐indol‐3-yl)propan‐1‐one (7)

Yield: 491 mg (74%) as a transparent crystal (re-crystallization from MeOH/EtOAc (1:1)). ^1^H NMR (400 MHz, DMSO-*d*_6_) δ 10.76 (br s, 1H), 7.53 (d, *J* = 8.0 Hz, 1H), 7.32 (d, *J* = 8.0 Hz, 1H), 7.15 (d, *J* = 2.0 Hz, 1H), 7.06 (td, *J* = 8.0, 0.8 Hz, 1H), 6.97 (td, *J* = 8.0, 0.8 Hz, 1H), 6.42 (s, 1H), 3.67–3.64 (m, 2H), 3.62–3.60 (m, 2H), 3.53–3.50 (m, 2H), 3.46–3.44 (m, 2H), 2.95 (t, *J* = 7.2 Hz, 2H), 2.71 (t, *J* = 7.2 Hz, 2H), 2.22 (s, 6H). ^13^C NMR (100 MHz, DMSO-*d*_6_) δ 170.8, 166.8 (2 × C), 160.8, 136.2, 127.1, 122.5, 120.9, 118.3, 118.2, 113.8, 111.3, 109.1, 44.8, 43.4, 43.1, 40.9, 33.4, 23.7 (2 × CH_3_), 20.6. HRMS-ESI (*m*/*z*) [M + H]^+^ calcd for C_21_H_26_N_5_O, 364.2132; found, 364.2131 (Δ = 0.2 ppm). [M + Na]^+^ calcd for C_21_H_25_N_5_NaO, 386.1951; found, 386.1949 (Δ = 0.5 ppm). HPLC (1): t = 10.2 min, 98.4% purity. HPLC (3): t = 15.6 min, 98.4% purity.

#### 1‐[4‐(4,6‐Dimethylpyrimidin‐2‐yl)piperazin‐1‐yl]‐4‐(1*H*‐indol‐3‐yl)butan‐1‐one (8)

Yield: 411 mg (66%) as an off-white crystal (re-crystallization from MeOH/EtOAc (2:1)). ^1^H NMR (400 MHz, DMSO-*d*_6_) δ 10.75 (br s, 1H), 7.52 (d, *J* = 8.0 Hz, 1H), 7.32 (d, *J* = 8.0 Hz, 1H), 7.11 (d, *J* = 2.4 Hz, 1H), 7.05 (td, *J* = 8.0, 0.8 Hz, 1H), 6.96 (td, *J* = 8.0, 0.8 Hz, 1H), 6.43 (s, 1H), 3.70–3.67 (m, 4H), 3.53–3.50 (m, 2H), 3.46–3.44 (m, 2H), 2.72 (t, *J* = 7.2 Hz, 2H), 2.41 (t, *J* = 7.2 Hz, 2H), 2.23 (s, 6H), 1.89 (quintet, *J* = 7.2 Hz, 2H). ^13^C NMR (100 MHz, DMSO-*d*_6_) δ 170.8, 166.8 (2 × C), 160.1, 136.3, 127.2, 122.3, 120.8, 118.3, 118.1, 114.2, 111.3, 109.1, 44.7, 43.5, 43.1, 40.9, 32.1, 25.6, 24.3, 23.7 (2 × CH_3_). HRMS-ESI (*m*/*z*) [M + H]^+^ calcd for C_22_H_28_N_5_O, 378.2288; found, 378.2295 (Δ = -1.8 ppm). [M + Na]^+^ calcd for C_22_H_27_N_5_NaO, 400.2108; found, 400.2110 (Δ = -0.7 ppm). HPLC (1): t = 11.3 min, 99.7% purity. HPLC (3): t = 16.3 min, 99.1% purity.

#### 3‐[4‐(4,6‐Dimethylpyrimidin‐2‐yl)piperazine‐1‐carbonyl]‐4‐methoxy‐1*H*‐indole (13)

Yield: 219 mg, (69%) as a brown solid. ^1^H NMR (400 MHz, DMSO-*d*_6_) δ 11.40 (br s, 1H), 7.37 (d, *J* = 1.2 Hz, 1H), 7.09–7.02 (m, 2H), 6.55 (d, *J* = 7.2 Hz, 1H), 6.42 (s, 1H), 3.80–3.68 (2 × br s, 11H), 2.22 (s, 6H). ^13^C NMR (100 MHz, DMSO-*d*_6_) δ 166.8 (2 × C), 166.3, 161.1, 152.8, 136.9, 124.1, 122.8, 115.0, 110.4, 109.1, 105.1, 100.1, 55.1, 43.1 (2 × CH_2_), 40.1–38.9 (2 × CH_2_), 23.7 (2 × CH_3_). HRMS-ESI (*m*/*z*) [M + H]^+^ calcd for C_20_H_24_N_5_O_2_, 366.1925; found, 366.1925 (Δ = -0.2 ppm). [M + Na]^+^ calcd for C_20_H_23_N_5_NaO_2_, 388.1744; found, 388.1739 (Δ = 1.3 ppm). HPLC (1): t = 8.7 min, 99.2% purity. HPLC (3): t = 15.4 min, 99.3% purity.

#### 3‐[4‐(4,6‐Dimethylpyrimidin‐2‐yl)piperazine‐1‐carbonyl]‐5‐methoxy‐1*H*‐indole (14)

Yield: 206 mg, (47%) as light yellow shiny transparent crystals (re-crystallization from MeOH/EtOAc (1:1)). ^1^H NMR (400 MHz, DMSO-*d*_6_) δ 11.50 (br s, 1H), 7.68 (s, 1H), 7.33 (d, *J* = 8.8 Hz, 1H), 7.21 (d, *J* = 2.4 Hz, 1H), 6.80 (dd, *J* = 8.8, 2.4 Hz, 1H), 6.44 (s, 1H), 3.83–3.80 (m, 4H), 3.76 (s, 3H), 3.71–3.69 (m, 4H), 2.24 (s, 6H). ^13^C NMR (100 MHz, DMSO-*d*_6_) δ 166.8 (2 × C), 165.9, 161.1, 154.3, 130.7, 128.5, 126.8, 112.7, 112.3, 109.3, 109.1, 101.8, 55.3, 43.6 (2 × CH_2_), 40.1–38.9 (2 × CH_2_), 23.7 (2 × CH_3_). HRMS-ESI (*m*/*z*) [M + H]^+^ calcd for C_20_H_24_N_5_O_2_, 366.1925; found, 366.1930 (Δ = -1.4 ppm). [M + Na]^+^ calcd for C_20_H_23_N_5_NaO_2_, 388.1744; found, 388.1742 (Δ = 0.4 ppm). HPLC (1): t = 8.4 min, 99.6% purity. HPLC (3): t = 15.2 min, 99.0% purity.

#### 1‐[4‐(4,6‐Dimethylpyrimidin‐2‐yl)piperazin‐1‐yl]‐2‐(5‐fluoro‐2‐methyl‐1*H*‐indol‐3‐yl)ethan‐1‐one (15)

Yield: 133 mg, (44%) as transparent colourless crystals (re-crystallization from MeOH/EtOAc (6:5)). ^1^H NMR (400 MHz, DMSO-*d*_6_) δ 10.92 (br s, 1H), 7.22–7.19 (m, 2H), 6.79 (td, *J* = 9.2, 2.4 Hz, 1H), 6.41 (s, 1H), 3.73 (s, 2H), 3.63–3.62 (m, 2H), 3.52 (s, 6H), 2.33 (s, 3H), 2.21 (s, 6H). ^13^C NMR (100 MHz, DMSO-*d*_6_) δ 169.4, 166.8 (2 × C), 161.0, 156.7 (d, *J* = 228.9 Hz, *C*-F), 135.0, 131.7, 128.6 (d, *J* = 10.2 Hz), 111.1 (d, *J* = 9.5 Hz), 109.1, 107.7 (d, *J* = 25.6 Hz, F-C-*C*-H), 104.5 (d, *J* = 4.3 Hz), 102.7 (d, *J* = 23.3 Hz, F-C-*C*-H), 45.2, 43.3, 43.2, 41.2, 29.7, 23.7 (2 × CH_3_), 11.6. HRMS-ESI (*m*/*z*) [M + H]^+^ calcd for C_21_H_25_FN_5_O, 382.2038; found, 382.2042 (Δ = -1.2 ppm). [M + Na]^+^ calcd for C_21_H_24_FN_5_NaO, 404.1857; found, 404.1852 (Δ = 1.2 ppm). HPLC (1): t = 10.1 min, 99.7% purity. HPLC (3): t = 15.8 min, 98.7% purity.

#### 5‐Chloro‐3‐[4‐(4,6‐dimethylpyrimidin‐2‐yl)piperazine‐1‐carbonyl]‐1*H*‐indole (16)

Yield: 137 mg, (43%) as pink crystals (re-crystallization from MeOH/EtOAc 1:1). ^1^H NMR (400 MHz, DMSO-*d*_6_) δ 11.83 (br s, 1H), 7.83 (s, 1H), 7.76 (dd, *J* = 2.0, 0.4 Hz, 1H), 7.47 (dd, *J* = 8.8, 0.4 Hz, 1H), 7.17 (dd, *J* = 8.4, 2.0 Hz, 1H), 6.44 (s, 1H), 3.82–3.80 (m, 4H), 3.73–3.70 (m, 4H), 2.24 (s, 6H). ^13^C NMR (100 MHz, DMSO-*d*_6_) δ 167.3 (2 × C), 165.5, 161.6, 134.7, 130.0, 128.1, 125.4, 122.5, 120.1, 114.0, 109.61, 109.57, 43.9 (2 × CH_2_), 40.6–39.3 (2 × CH_2_), 24.2 (2 × CH_3_). HRMS-ESI (*m*/*z*) [M + H]^+^ calcd for C_19_H_21_ClN_5_O, 370.1429; found, 370.1433 (Δ = -0.9 ppm). [M + Na]^+^ calcd for C_19_H_20_ClN_5_NaO, 392.1249; found, 392.1245 (Δ = 0.9 ppm). HPLC (1): t = 10.9 min, 99.1% purity. HPLC (3): t = 16.6 min, 98.1% purity.

#### 2‐(5‐Bromo‐7‐fluoro‐2‐methyl‐1*H*‐indol‐3‐yl)‐1‐[4‐(4,6-dimethylpyrimidin‐2‐yl)piperazin‐1‐yl]ethan‐1‐one (17)

Yield: 130 mg, (50%) as dark brown crystals (re-crystallization from MeOH/EtOAc 9:1). ^1^H NMR (400 MHz, DMSO-*d*_6_) δ 11.53 (br s, 1H), 7.48 (d, *J* = 1.2 Hz, 1H), 7.04 (dd, *J* = 10.4, 1.2 Hz, 1H), 6.43 (s, 1H), 3.75 (s, 2H), 3.66–3.64 (m, 2H), 3.61–3.59 (m, 2H), 3.55–3.53 (m, 4H), 2.34 (s, 3H), 2.25 (s, 6H). ^13^C NMR (100 MHz, DMSO-*d*_6_) δ 169.5, 167.3 (2 × C), 161.4, 148.5 (d, *J* = 245.7 Hz, *C*-F), 136.5, 133.8 (d, *J* = 7.3 Hz), 122.1 (d, *J* = 13.2 Hz, F-C-*C*-NH), 117.3 (d, *J* = 2.9 Hz), 109.8 (d, *J* = 8.0 Hz), 109.6, 108.7 (d, *J* = 20.4 Hz, F-C-*C*-H), 105.9 (d, *J* = 2.2 Hz), 45.5, 43.8, 43.6, 41.7, 29.7, 24.1 (2 × CH_3_), 11.9. HRMS-ESI (*m*/*z*) [M + H]^+^ calcd for C_21_H_24_BrFN_5_O, 460.1143; found, 460.1132 (Δ = 2.3 ppm). [M + Na]^+^ calcd for C_21_H_23_BrFN_5_NaO, 482.0962; found, 482.0950 (Δ = 2.5 ppm). HPLC (1): t = 13.3 min, 95.9% purity. HPLC (3): t = 17.2 min, 96.5% purity.

#### 1‐[4‐(4,6‐Dimethylpyrimidin‐2‐yl)piperazin‐1‐yl]‐2‐(1*H*‐indol‐3‐yl)ethan‐1‐one (18)

Yield: 414 mg, (46%) as white crystals (re-crystallization from EtOH/EtOAc 1:1). ^1^H NMR (400 MHz, DMSO-*d*_6_) δ 10.90 (br s, 1H), 7.58 (d, *J* = 7.6 Hz, 1H), 7.34 (d, *J* = 8.4 Hz, 1H), 7.24 (d, *J* = 2.0 Hz, 1H), 7.07 (td, *J* = 7.4, 0.8 Hz, 1H), 6.97 (td, *J* = 7.4, 0.8 Hz, 1H), 6.41 (s, 1H), 3.81 (s, 2H), 3.66–3.63 (m, 2H), 3.57 (s, 4H), 3.53–3.51 (m, 2H), 2.21 (s, 6H). ^13^C NMR (100 MHz, DMSO-*d*_6_) δ 169.4, 166.8 (2 × C), 161.0, 136.1, 127.1, 123.5, 121.1, 118.8, 118.4, 111.3, 109.1, 108.1, 45.4, 43.4, 43.1, 41.1, 30.9, 23.7 (2 × CH_3_). HRMS-ESI (*m*/*z*) [M + H]^+^ calcd for C_20_H_24_N_5_O, 350.1975; found, 350.1980 (Δ = -1.4 ppm). [M + Na]^+^ calcd for C_20_H_23_N_5_NaO, 372.1795; found, 372.1794 (Δ = 0.2 ppm). HPLC (1): t = 9.2 min, 97.5% purity. HPLC (3): t = 15.3 min, 99.2% purity.

#### 1‐[4‐(4,6‐Dimethylpyrimidin‐2‐yl)piperazin‐1‐yl]‐2‐(6‐fluoro‐1*H*‐indol‐3‐yl)ethan‐1‐one (19)

Yield: 179 mg, (57%) as light brown crystals (re-crystallization from MeOH/EtOAc 8:5). ^1^H NMR (400 MHz, DMSO-*d*_6_) δ 10.97 (br s, 1H), 7.56 (dd, *J* = 8.8, 5.6 Hz, 1H), 7.24 (d, *J* = 2.0 Hz, 1H), 7.11 (dd, *J* = 10.0, 2.4 Hz, 1H), 6.86–6.81 (m, 1H), 6.42 (s, 1H), 3.81 (s, 2H), 3.66–3.63 (m, 2H), 3.58 (s, 4H), 3.53–3.50 (m, 2H), 2.21 (s, 6H). ^13^C NMR (100 MHz, DMSO-*d*_6_) δ 169.8, 167.2 (2 × C), 161.5, 159.3 (d, *J* = 232.6 Hz, *C*-F), 136.4 (d, *J* = 12.4 Hz), 124.6 (d, *J* = 3.7 Hz), 124.5, 120.3 (d, *J* = 10.2 Hz), 109.6, 108.9, 107.3 (d, *J* = 24.1 Hz, F-C-*C*-NH), 97.7 (d, *J* = 24.8 Hz, F-C-*C*-NH), 45.8, 43.9, 43.6, 41.6, 31.2, 24.1 (2 × CH_3_). HRMS-ESI (*m*/*z*) [M + H]^+^ calcd for C_20_H_23_FN_5_O, 368.1881; found, 368.1879 (Δ = 0.6 ppm). [M + Na]^+^ calcd for C_20_H_22_FN_5_NaO, 390.1701; found, 390.1689 (Δ = 2.9 ppm). HPLC (1): t = 9.6 min, 99.8% purity. HPLC (3): t = 15.6 min, 99.0% purity.

#### 3‐[4‐(4,6‐Dimethylpyrimidin‐2‐yl)piperazine‐1‐carbonyl]‐7‐methoxy‐1*H*‐indole (20)

Yield: 215 mg, (57%) as an off-white solid.^1^H NMR (400 MHz, DMSO-*d*_6_) δ 11.75 (br s, 1H), 7.59 (d, *J* = 2.4 Hz, 1H), 7.29 (d, *J* = 7.2 Hz, 1H), 7.02 (t, *J* = 8.0 Hz, 1H), 6.73 (d, *J* = 7.6 Hz, 1H), 6.44 (s, 1H), 3.93 (s, 3H), 3.81–3.78 (m, 4H), 3.69–3.66 (m, 4H), 2.24 (s, 6H). ^13^C NMR (100 MHz, DMSO-*d*_6_) δ 166.8 (2 × C), 165.8, 161.1, 146.3, 127.6, 127.4, 125.9, 120.9, 112.9, 110.3, 109.1, 102.4, 55.3, 43.5 (2 × CH_2_), 40.1–38.9 (2 × CH_2_), 23.7 (2 × CH_3_). HRMS-ESI (*m*/*z*) [M + H]^+^ calcd for C_20_H_24_N_5_O_2_, 366.1925; found, 366.1920 (Δ = 1.2 ppm). [M + Na]^+^ calcd for C_20_H_23_N_5_NaO_2_, 388.1744; found, 388.1733 (Δ = 2.9 ppm). HPLC (1): t = 9.5 min, 99.8% purity. HPLC (3): t = 15.9 min, 99.3% purity.

#### 2‐[4‐(4,6‐Dimethylpyrimidin‐2‐yl)piperazine‐1‐carbonyl]‐1*H*‐indole (21)

Yield: 241 mg, (23%) as clear crystals (re-crystallization from MeOH/EtOAc 1:1). ^1^H NMR (400 MHz, DMSO-*d*_6_) δ 11.60 (br s, 1H), 7.62 (dd, *J* = 8.0, 0.4 Hz, 1H), 7.43 (dd, *J* = 8.0, 0.4 Hz, 1H), 7.21–7.17 (m, 1H), 7.07–7.03 (m, 1H), 6.86 (dd, *J* = 2.0, 0.8 Hz, 1H), 6.46 (s, 1H), 3.84 (br s, 8H), 2.25 (s, 6H). ^13^C NMR (100 MHz, DMSO-*d*_6_) δ 166.9 (2 × C), 162.2, 161.0, 136.0, 129.8, 126.9, 123.3, 121.4, 119.8, 112.1, 109.2, 104.3, 43.4 (2 × CH_2_), 40.1–38.9 (2 × CH_2_), 23.7 (2 × CH_3_). HRMS-ESI (*m*/*z*) [M + H]^+^ calcd for C_19_H_22_N_5_O, 336.1819; found, 336.1821 (Δ = -0.8 ppm). [M + Na]^+^ calcd for C_19_H_21_N_5_NaO, 358.1638; found, 358.1634 (Δ = 1.1 ppm). HPLC (1): t = 11.3 min, 95.0% purity. HPLC (3): t = 16.5 min, 99.3% purity.

#### 2‐[4‐(4,6‐Dimethylpyrimidin‐2‐yl)piperazine‐1‐carbonyl]‐5‐fluoro‐1*H*‐indole (22)

Yield: 31 mg, (16%) as white silky crystals (re-crystallization from EtOH/EtOAc 1:1). ^1^H NMR (400 MHz, DMSO-*d*_6_) δ 11.72 (br s, 1H), 7.43 (dd, *J* = 8.8, 4.4 Hz, 1H), 7.37 (dd, *J* = 9.6, 2.4 Hz, 1H), 7.06 (td, *J* = 9.6, 2.4 Hz, 1H), 6.84 (s, 1H), 6.46 (s, 1H), 3.84 (br s, 8H), 2.25 (s, 6H). ^13^C NMR (100 MHz, DMSO-*d*_6_) δ 166.8 (2 × C), 161.9, 161.0, 157.1 (d, *J* = 231.1 Hz, *C*-F), 132.7, 131.6, 126.9 (d, *J* = 10.2 Hz), 113.3 (d, *J* = 9.4 Hz), 111.9 (d, J = 26.2 Hz, F-C-*C*-H), 109.2, 105.5 (d, J = 22.6 Hz, F-C-*C*-H), 104.2 (d, J = 5.1 Hz), 43.3 (2 × CH_2_), 40.1–38.9 (2 × CH_2_), 23.7 (2 × CH_3_). HRMS-ESI (*m*/*z*) [M + H]^+^ calcd for C_19_H_21_FN_5_O, 354.1725; found, 354.1720 (Δ = 0.4 ppm). [M + Na]^+^ calcd for C_19_H_20_FN_5_NaO, 376.1544; found, 376.1537 (Δ = 0.7 ppm). HPLC (1): t = 11.7 min, 98.0% purity. HPLC (3): t = 16.6 min, 99.7% purity.

#### 2‐[4‐(4,6‐Dimethylpyrimidin‐2‐yl)piperazine‐1‐carbonyl]‐5‐methyl‐1H‐indole (23)

Yield: 70 mg, (35%) as brown crystals (re-crystallization from MeOH/EtOAc 8:5). ^1^H NMR (400 MHz, DMSO-*d*_6_) δ 11.46 (br s, 1H), 7.38 (d, *J* = 0.8 Hz, 1H), 7.32 (d, *J* = 8.4 Hz, 1H), 7.02 (dd, *J* = 8.4, 1.6 Hz, 1H), 6.76 (d, *J* = 1.6 Hz, 1H), 6.46 (s, 1H), 3.84 (br s, 8H), 2.37 (s, 3H), 2.25 (s, 6H). ^13^C NMR (100 MHz, DMSO-*d*_6_) δ 167.3 (2 × C), 162.7, 161.5, 134.8, 130.2, 128.8, 127.6, 125.6, 121.1, 112.3, 109.7, 104.3, 43.8 (2 × CH_2_), 40.6–9.3 (2 × CH_2_), 24.2 (2 × CH_3_), 21.6. HRMS-ESI (*m*/*z*) [M + H]^+^ calcd for C_20_H_24_N_5_O, 350.1975; found, 350.1970 (Δ = 1.5 ppm). [M + Na]^+^ calcd for C_20_H_23_N_5_NaO, 372.1795; found, 372.1785 (Δ = 2.7 ppm). HPLC (1): t = 13.1 min, 98.9% purity. HPLC (3): t = 17.1 min, 98.3% purity.

#### 2‐[4‐(4,6‐Dimethylpyrimidin‐2‐yl)piperazine‐1‐carbonyl]‐5‐methoxy‐1*H*‐indole (24)

Yield: 139 mg, (36%) as shiny, colourless crystals (re-crystallization from MeOH/EtOAc 8:5). ^1^H NMR (400 MHz, DMSO-*d*_6_) δ 11.45 (br s, 1H), 7.32 (d, *J* = 8.8 Hz, 1H), 7.07 (d, *J* = 2.4 Hz, 1H), 6.85 (dd, *J* = 8.8, 2.4 Hz, 1H), 6.76 (d, *J* = 1.2 Hz, 1H), 6.46 (s, 1H), 3.83 (br s, 8H), 3.76 (s, 3H), 2.25 (s, 6H). ^13^C NMR (100 MHz, DMSO-*d*_6_) δ 166.9 (2 × C), 162.2, 161.1, 153.8, 131.3, 130.2, 127.2, 114.4, 113.0, 109.2, 104.1, 102.0, 55.3, 43.3 (2 × CH_2_), 40.1–38.9 (2 × CH_2_), 23.7 (2 × CH_3_). HRMS-ESI (*m*/*z*) [M + H]^+^ calcd for C_20_H_24_N_5_O_2_, 366.1925; found, 366.1925 (Δ = -0.1 ppm). [M + Na]^+^ calcd for C_20_H_23_N_5_NaO_2_, 388.1744; found, 388.1733 (Δ = 2.7 ppm). HPLC (1): t = 10.6 min, 97.2% purity. HPLC (3): t = 16.2 min, 99.2% purity.

#### {2‐[4‐(4,6‐Dimethylpyrimidin‐2‐yl)piperazine‐1‐carbonyl]‐1*H*‐indol‐7‐yl}azinic acid (25)

Yield: 137 mg, (36%) as a light yellow solid. ^1^H NMR (400 MHz, DMSO-*d*_6_) δ 11.77 (br s, 1H), 8.20 (d, *J* = 8.0 Hz, 1H), 8.15 (d, *J* = 8.0 Hz, 1H), 7.32 (t, *J* = 8.0 Hz, 1H), 7.04 (d, *J* = 2.4 Hz, 1H), 6.46 (s, 1H), 3.84 (br s, 4H), 3.71 (br s, 4H), 2.24 (s, 6H). ^13^C NMR (100 MHz, DMSO-*d*_6_) δ 166.8 (2 × C), 161.5, 161.0, 133.9, 133.0, 131.1, 130.0, 128.1, 120.2, 119.7, 109.3, 104.5, 40.1–38.9 (4 × CH_2_), 23.7 (2 × CH_3_). HRMS-ESI (*m*/*z*) [M + H]^+^ calcd for C_19_H_21_N_6_O_3_, 381.1670; found, 381.1662 (Δ = 1.9 ppm). [M + Na]^+^ calcd for C_19_H_20_N_6_NaO_3_, 403.1489; found, 403.1480 (Δ = 2.2 ppm). HPLC (x): t = 11.7 min, 97.3% purity. HPLC (3): t = 16.8 min, 99.0% purity.

#### General procedure for the reduction reactions to obtain compounds 9–12

To a slurry of LiAlH_4_ in anhydrous THF at 0°C was added dropwise by syringe the solution of the starting carbonyl compound in anhydrous THF, and the mixture was warmed to 23°C with stirring for 4~21.5 h. At 0°C, the following sequence of dropwise addition was performed with continuous stirring: H_2_O, 15% aqueous NaOH. The white sticky part was removed by filtration, and the filtrate was concentrated under reduced pressure. The crude product was purified by flash chromatography (EtOAc/hexane).

#### 3‐{[4‐(4,6‐Dimethylpyrimidin‐2‐yl)piperazin‐1‐yl]methyl}‐1*H*‐indole (9)

Yield: 391 mg, (76%) as a yellow solid (EtOAc/hexane 1:1). ^1^H NMR (400 MHz, DMSO-*d*_6_) δ 10.93 (br s, 1H), 7.64 (d, *J* = 7.6 Hz, 1H), 7.35 (d, *J* = 7.6 Hz, 1H), 7.23 (d, *J* = 2.4 Hz, 1H), 7.07 (td, *J* = 6.8, 1.2 Hz, 1H), 6.98 (td, *J* = 6.8, 1.2 Hz, 1H), 6.36 (s, 1H), 3.71–3.68 (m, 4H), 3.65 (s, 2H), 2.43–2.40 (m, 4H), 2.20 (s, 6H). ^13^C NMR (100 MHz, DMSO-*d*_6_) δ 166.6 (2 × C), 161.1, 136.3, 127.7, 124.7, 121.0, 119.0, 118.5, 111.4, 110.6, 108.6, 53.2, 52.5 (2 × CH_2_), 43.4 (2 × CH_2_), 23.7 (2 × CH_3_). HRMS-ESI (*m*/*z*) [M + H]^+^ calcd for C_19_H_24_N_5_, 322.2026; found, 322.2029 (Δ = -1.0 ppm). [M + Na]^+^ calcd for C_19_H_23_N_5_Na, 344.1846; found, 344.1845 (Δ = 0.3 ppm). HPLC (1): t = 8.5 min, 100% purity. HPLC (3): t = 14.6 min, 97% purity.

#### 3‐{2‐[4‐(4,6‐Dimethylpyrimidin‐2‐yl)piperazin‐1‐yl]ethyl}‐2‐methyl‐1*H*‐indole (10)

Yield: 546 mg, (95%) as a yellow solid (EtOAc/hexane 6:1). ^1^H NMR (400 MHz, DMSO-*d*_6_) δ 10.67 (br s, 1H), 7.39 (d, *J* = 7.6 Hz, 1H), 7.21 (d, *J* = 7.6 Hz, 1H), 6.96 (td, *J* = 7.2, 1.2 Hz, 1H), 6.91 (td, *J* = 7.2, 1.2 Hz, 1H), 6.39 (s, 1H), 3.74–3.72 (m, 4H), 2.83–2.79 (m, 2H), 2.46–2.44 (m, 6H), 2.32 (s, 3H), 2.22 (s, 6H). ^13^C NMR (100 MHz, DMSO-*d*_6_) δ 166.6 (2 × C), 161.2, 135.1, 131.6, 128.2, 119.8, 118.0, 117.3, 110.3, 108.7, 108.2, 59.1, 52.7 (2 × CH_2_), 43.4 (2 × CH_2_), 23.7 (2 × CH_3_), 21.5, 11.2. HRMS-ESI (*m*/*z*) [M + H]^+^ calcd for C_21_H_28_N_5_, 350.2339; found, 350.2345 (Δ = -1.6 ppm). [M + Na]^+^ calcd for C_21_H_27_N_5_Na, 372.2159; found, 372.2166 (Δ = -2.0 ppm). HPLC (2): t = 9.0 min, 96.7% purity. HPLC (3): t = 14.5 min, 96.3% purity.

#### 3‐{3‐[4‐(4,6‐Dimethylpyrimidin‐2‐yl)piperazin‐1‐yl]propyl}‐1*H*‐indole (11)

Yield: 700 mg, (97%) as a white solid (EtOAc/hexane 1:1). ^1^H NMR (400 MHz, DMSO-*d*_6_) δ 10.74 (br s, 1H), 7.51 (d, *J* = 7.6 Hz, 1H), 7.32 (d, *J* = 7.6 Hz, 1H), 7.11 (d, *J* = 2 Hz, 1H), 7.05 (td, *J* = 7.2, 1.2 Hz, 1H), 6.96 (td, *J* = 7.2, 1.2 Hz, 1H), 6.38 (s, 1H), 3.71–3.69 (m, 4H), 2.73–2.69 (m, 2H), 2.40–2.34 (m, 6H), 2.21 (s, 6H), 1.83 (quintet, *J* = 7.6 Hz, 2H). ^13^C NMR (100 MHz, DMSO-*d*_6_) δ 166.6 (2 × C), 161.2, 136.3, 127.2, 122.2, 120.8, 118.3, 118.1, 114.4, 111.3, 108.7, 57.8, 52.8 (2 × CH_2_), 43.3 (2 × CH_2_), 27.2, 23.7 (2 × CH_3_), 22.5. HRMS-ESI (*m*/*z*) [M + H]^+^ calcd for C_21_H_28_N_5_, 350.2339; found, 350.2346 (Δ = -1.9 ppm). [M + Na]^+^ calcd for C_21_H_27_N_5_Na, 372.2159; found, 372.2166 (Δ = -2.0 ppm). HPLC (2): t = 9.0 min, 100% purity. HPLC (3): t = 14.4 min, 96.2% purity.

#### 3‐{4‐[4‐(4,6‐Dimethylpyrimidin‐2‐yl)piperazin‐1‐yl]butyl}‐1*H*‐indole (12)

Yield: 510 mg, (97%) as a white solid (EtOAc/hexane 4:6). ^1^H NMR (400 MHz, DMSO-*d*_6_) δ 10.72 (br s, 1H), 7.50 (d, *J* = 7.6 Hz, 1H), 7.32 (d, *J* = 7.6 Hz, 1H), 7.10 (d, *J* = 2.4 Hz, 1H), 7.05 (td, *J* = 6.8, 0.8 Hz, 1H), 6.95 (td, *J* = 6.8, 0.8 Hz, 1H), 6.38 (s, 1H), 3.69–3.66 (m, 4H), 2.70 (t, *J* = 7.2 Hz, 2H), 2.37–2.31 (m, 6H), 2.21 (s, 6H), 1.67 (quintet, *J* = 7.6 Hz, 2H), 1.52 (quintet, *J* = 7.6 Hz, 2H). ^13^C NMR (100 MHz, DMSO-*d*_6_) δ 166.6 (2 × C), 161.2, 136.3, 127.2, 122.1, 120.7, 118.3, 118.0, 114.6, 111.3, 108.7, 57.8, 52.7 (2 × CH_2_), 43.3 (2 × CH_2_), 27.7, 26.2, 24.5, 23.7 (2 × CH_3_). HRMS-ESI (*m*/*z*) [M + H]^+^ calcd for C_22_H_30_N_5_, 364.2496; found, 364.2497 (Δ = -0.4 ppm). [M + Na]^+^ calcd for C_22_H_29_N_5_Na, 386.2315; found, 386.2318 (Δ = -0.8 ppm). HPLC (2): t = 9.3 min, 100% purity. HPLC (3): t = 14.7 min, 98.5% purity.

### Biological evaluations

#### Membrane preparation

A two-step procedure was adopted to prepare membrane for radioligand binding from cells stably transfected with human adenosine receptor subtypes (hA_1_, hA_2A_ and hA_3_ expressed on CHO cells) [41]. Firstly, cell fragments and nuclei were removed by using low-speed centrifugation (1,000 *g* for 10 min). After that, crude membrane fraction from supernatant was then sedimented at 100,000 *g* for 30 min. The membrane pellet was subsequently re-suspended in the specific binding buffer, frozen in liquid nitrogen and stored at -80°C. For measurement of adenylyl cyclase activity in the hA_2B_ receptors, one-step centrifugation procedure was used; the homogenate was sedimented for 30 min at 54,000 *g*. The so-obtained crude membrane pellet was re-suspended in 50 mM Tris/HCl, pH 7.4 and used for the adenylyl cyclase assay immediately.

#### Binding assays for hA_1_, hA_2A_, hA_3_ adenosine receptors

In accordance with procedures described previously [41–43], competition binding experiment for human A_1_ adenosine receptors was carried out for 3 h at 25°C in 200 μL of buffer containing 1 nM [^3^H]CCPA (K_D_ = 0.61 nM), 0.2 U/mL adenosine deaminase, 20 μL of diluted membranes (50 μg of protein/assay) in 50 mM Tris/HCl, pH 7.4 and test compounds in different concentrations. Non-specific binding was determined using theophylline 1 mM. In a similar manner, binding of [^3^H]NECA to CHO cells transfected with hA_2A_ adenosine receptors was performed. A mixture of protein with a concentration of 50 μg/assay in buffer, 10 nM [^3^H]NECA (K_D_ = 20 nM) and test compound in different concentrations were incubated for 3 h at 25°C. Non-specific binding was determined using *N*^6^-*R*-phenylisopropyl adenosine (R-PIA) 100 μM. ^41^ The binding of [^3^H]HEMADO to CHO cells transfected with hA_3_ adenosine receptors was carried out as described earlier [44]. The binding experiment was carried out for 3 h at 25°C in the buffer solution containing 1 nM [^3^H]HEMADO (K_D_ = 1.1 nM), 50 μg membrane protein in 50 mM Tris-HCl, 1 mM ethylenediaminetetraacetic acid (EDTA), 10 mM MgCl_2_, pH 8.25 and test compound in different concentrations. Non-specific binding was determined using R-PIA 100 μM.

The assay mixture was then filtered through the built-in filter at the bottom of the 96-well microplate filtration system (Millipore Multiscreen MAFC) and washed three times with 200 μL of cold buffer. After addition of 20 μL of scintillator to the dried filter plates, the filter bound radioactivity was counted on a Wallac Micro-Beta counter. All K_*i*_ values were calculated by non-linear curve fitting with the program SCTFIT [45].

#### Adenylyl cyclase activity assay for hA_2B_ adenosine receptors

Due to the lack of a useful high-affinity radioligand for A_2B_ receptors, the potency of test compounds at hA_2B_ receptor was determined in adenylyl cyclase experiments as described previously [41,42]. Concentration-dependent inhibition of NECA-stimulated adenylyl cyclase (stimulation with 5 μM of NECA, EC_50_ = 2.4 μM) caused by test compounds was measured in membranes from CHO cells stably transfected with the hA_2B_ adenosine receptors. The membranes were incubated with about 150,000 cpm of [α-^32^P]adenosine triphosphate ([α-^32^P]ATP) and test compounds in different concentrations for 20 min. A non-linear regression analysis was applied to calculate the IC_50_ of the adenylyl cyclase activity assay. From the IC_50_ values, *K*_i_ values were then calculated using the Cheng and Prusoff equation [46].

#### Dopamine D_2_ receptor proteopolymersomes

Polymersomes (ABA triblock-copolymer and BD21 diblock-copolymer: ABA stands for PMOXA-PDMS-PMOXA; A = PMOXA = poly(2-methyloxazoline); B = PDMS = poly(dimethylsiloxane) and BD21 stands for [PBd]22-b-[PEO]13; PBd/PEO = polybutadiene/polyethylene oxide) preparation, cloning and *in vitro* synthesis of dopamine D_2_ receptor, and purification of proteopolymersomes were performed following previously described procedures [47]. For the replacement assay, ABA-polymersomes were covalently attached to an amino-functionalized glass slide, which was then treated with isopropanol, ultrapure water and a N_2_-stream. The slide was cut into small chips and each chip was treated with the ethanolic solution of the mixture containing tetrazol(4-(2-phenyl-2*H*-tetrazol-5-yl)benzoic acid, *N*-hydroxysuccinimide and *N*-(3-dimethylaminopropyl)-N’-ethylcarbodiimidehydrochloride, and then incubated for one hour. ABA with 10% methacrylate was dispensed onto the polydimethylsiloxane (PDMS) stamps and incubated for one hour. The photoinducible 1,3-dipolar cycloaddition between the tetrazole and the methacrylate functional group on the polymersomes was induced by 15 min incubation under UV light (254 nm). The chips were incubated with a 30 μM BODIPY-NAPS solution in Tris-MgCl_2_-NaCl (TMN) buffer for 30 min in the dark at room temperature. Program ImageJ was used for determination of fluorescence intensities.

#### Adenylyl cyclase inhibition assay for dopamine D_2_ receptors

CHO cells transfected with D_2_ receptors were cultured and then serum starved overnight. Cells were treated with various concentrations of ligands (25 μL of 10X solution), stimulated with forskolin (0.5 μM) and 3-isobutyl-1-methylxanthine (IBMX) (0.5 mM), and incubated for 15 minutes. After lysis, cAMP content was determined by ELISA.

#### *Drosophila* models of PD

Fly lines for *24B*-Gal4 (muscle specific), *ddc*-Gal4 (dopaminergic neuron-specific), *elav*-Gal4 (pan-neuronal), UAS-mito-GFP, and UAS-dAMPK-KA were purchased from Bloomington *Drosophila* Stock Center. The *parkin*-null mutant flies were kind gifts from Chung J. and Cho K. S. (Korea Advanced Institute of Science and Technology, Daejeon, Korea). To generate transgenic mutant LRRK2 G2019S, cDNA containing a myc-tag at the C terminus was inserted into pUAST plasmid and microinjected into *Drosophila* embryos (BestGene). Sequencing of cloned products was performed before they were microinjected into the embryos. Climbing assays were performed according to a previously described method [48]. Briefly, 20 female adult flies from each group were randomly selected after being anaesthetised and placed in a vertical plastic column (length 25 cm; diameter 1.5 cm). Age-matched normal flies were used as controls. After a 2-hour recovery period from CO_2_ exposure, flies were gently tapped to the bottom of the column, and the number of flies that reached the top of column at 1 min was counted. Results are presented as mean ± SEM of the scores obtained from three independent experiments. To study the effect of compounds, flies were fed with cornmeal-agar medium supplemented with the DMSO solution of the compound immediately at post-eclosion (for *parkin*-null flies) or at day 35 onwards (for LRRK2 mutant flies) for a period of 25 days. Immunohistochemical analysis of whole-mount adult fly brains were prepared according to published protocols [36] and stained with rabbit anti-TH (1:300, Pel-Freez Biologicals) as primary antibody. The stained samples were viewed using an Olympus Fluoview Upright Confocal Microscope. DA neurons were quantified according to previously published method [36]. The size of mito-GFP puncta was measured using the ImageJ program and expressed as mean ± SD (n ≥ 10 DA neurons per experimental group). Statistical significance for all the quantitative data obtained were analyzed using one-way ANOVA with Tukey’s test HSD *post hoc test* (*p < 0.05, **p < 0.01).

#### Mutagenicity test

*In vitro* mutagenicity was performed using a modified Ames Test protocol according to manufacturer’s instructions (MolTox). *S*. *typhimurium* strains (TA98 and TA100) were grown from bacterial discs in Oxoid #2 nutrient broth at 37°C in a shaking incubator (~150 rpm) for about 10 h. The cultures were then measured for absorbance with a UV spectrophotometer at 660 nm and to be used at a density of approximately 1.0 to 1.2 absorbance units. For compound treatment, the top agar was melted in a hot water bath or microwave oven and 2 mL volumes were aliquoted into culture tubes. The tubes of agar were then maintained at 45°C for at least 30 to 45 min for temperature equilibration. 100 μL of test compounds at concentration of 1 mM were added to separate tubes containing top agar in duplicates. Additional tubes were set aside as negative (DMSO, methotrexate) and positive (4-NQO and 2-AA) controls. 4-NQO was reported to be a DNA damaging agent in the cell-based assay with no added exogenous metabolic enzymes [49], while 2-AA was reported to be metabolically activated into mutagens by various CYP isozymes [50]. Subsequently, 500 μL of S9 mix was introduced to each tube containing either controls or test compounds. The contents were immediately mixed and decanted onto Minimal Glucose (MG) Agar Plate and swirled to obtain an even distribution of plating mixture over the agar surface. After the agar was set, the plates were then incubated at 37°C for 48 h. Images of colonies were captured and counted with the aid of ImageJ software.

#### Cytotoxicity test

Immortalised hepatocyte and cardiomyocyte cell lines, TAMH (TGF-α overexpressing mouse hepatocytes) and HL-1 were used respectively as models for *in vitro* toxicity studies. Both cell lines were cultured in accordance with previously described methods [51,52]. Briefly, TAMH lines between passages 21–35 were grown in serum-free DMEM/Ham’s F12 (Invitrogen, Carlsbad, CA) supplemented 5 μg/mL insulin, 5 μg/mL transferrin, 5 ng/mL selenium (Collaborative Biomedical Products, 354351 Boston, MA), 100 nM dexamethasone, 10 mM nicotinamide and 0.1% v/v gentamicin (Invitrogen) and 0.12% sodium bicarbonate (Sigma 5671). For HL-1 cells, cell culture flasks were first coated with fibronectin/gelatin (25 μg of fibronectin in 2 mL of 0.02% gelatin in water per T25 flask (Sigma G1393 & F1141)) overnight at 37°C and the excess fluid was aspirated thereafter. Cells were then maintained in Claycomb medium (Sigma 51800C) supplemented with FBS (Sigma F2442), 2 mM L-glutamine (Sigma), 10 μM norepinephrine, 100 U/ml penicillin, 100 μg/ml streptomycin and 1X non-essential amino acids. Medium was changed every 24 h. All cultures were maintained in a humidified incubator with 5% carbon dioxide/95% air at 37°C and passaged at 70–90% confluence. TAMH cells were plated into 96-well plates at 12,000 cells per well whilst HL-1 at 15,000 cells per well and incubated overnight. The following day, test compounds were prepared from DMSO stock solutions and diluted to concentrations of 0.03 μM to 100 μM. Medium was aspirated and replaced with respective compound concentrations and incubated for another 24 h at 37°C (n = 6). Subsequently, Cell-Titer-Glo (Promega G7571) assay was performed according to manufacturer’s instructions. The cell-reagent mixture was transferred to a solid white flat-bottom 96-well plate (Greiner 655207) for luminescence reading. Luminescence was recorded with an integration time of 0.25 second with a Tecam Infinite® M200 Microplate reader. Data are expressed as percentage of viable cells compared to DMSO-treated controls. Semi-log graphs were plotted using GraphPad Prism (La Jolla, CA, USA).

#### Aqueous solubility studies

The 10 μL stock solution of the ligand (10 mM in DMSO) was added to the universal aqueous buffer (pH 7.4), and the mixture was sonicated. 300 μL of the turbid mixture was transferred to the 3 wells of MultiScreen HTS- PCF filter plate (Millipore Corp., Ireland), and the plate was covered and incubated with gentle shaking (250 rpm, 3 h) at room temperature (22.5 ± 2.5°C). After the period of incubation (3 h), the filter plate was placed on a vacuum manifold and the contents were filtered into a 96-well UV plate. After filtration, 200 μL of filtrate was transferred from each well to the PP vial. Absorbances of the solutions were quantified by HPLC-UV and read at 250 nm.

## Supporting information

S1 Table Number of collected A_2A_ antagonists and D_2_ agonists. (DOC)

S2 Table SVM-based virtual screening of PubChem & MDDR Databases. (DOC)

S3 Table Compound Identification Number (CID) and chemical structures of 172 hits. (DOC)

S1 Fig Results of Tanimoto-based similarity searching with Tc > 0.9. (DOC)

S2 Fig Ten clusters from dendrogram and cluster analysis. (DOC)

S3 Fig Design from cluster 1. (DOC)

S4 Fig Design from cluster 2. (DOC)

S5 Fig Design from cluster 3. (DOC)

S6 Fig Design from cluster 8. (DOC)

S7 Fig Design from cluster 9. (DOC)

S8 Fig Ten compounds that at least pass one model of A_2A_ and one model of D_2_. (DOC)

S9 Fig Number of His^+^-revertant colonies grown on the agar plates containing various chemicals and (A) TA98 strain without “S9 mix”, (B) TA98 strain with “S9 mix”, (C) TA100 strain without “S9 mix”, and (D) TA100 strain with “S9 mix”. Blank indicates spontaneously induced revertants without treatment with any drug or solvent.
(DOC)

S10 Fig Cell viability after treatment of TAMH (A-C) and HL-1 (D-F) cells with positive controls and compounds.
(DOC)
