# Supplementary material for: Discovery of indolylpiperazinylpyrimidines with dual-target profiles at adenosine A2A and dopamine D2 receptors for Parkinson's disease treatment
Source: PLoS One. 2018 Jan 5;13(1):e0188212. doi: 10.1371/journal.pone.0188212 (PMC5755735; doi:10.1371/journal.pone.0188212)
Supplement: S1 Table — (DOC) [file pone.0188212.s001.doc]

**S1 Table. Number of collected A_2A_ Antagonists and D_2_** Agonists

|  | A_2A_ antagonists | D_2_ agonists | A_2A_ antagonists & D_2_ agonists |
| --- | --- | --- | --- |
| Binding | 1,969 | 810 | 0 |
| Function | 418 | 332 | 0 |
