# Supplementary material for: Discovery of indolylpiperazinylpyrimidines with dual-target profiles at adenosine A2A and dopamine D2 receptors for Parkinson's disease treatment
Source: PLoS One. 2018 Jan 5;13(1):e0188212. doi: 10.1371/journal.pone.0188212 (PMC5755735; doi:10.1371/journal.pone.0188212)
Supplement: S2 Table — (DOC) [file pone.0188212.s002.doc]

**S2 Table. SVM-based virtual screening of PubChem & MDDR Databases**

|  | Source | SVM1 (binding) | SVM2 (function) | SVM3 (function + MDDR) | SVM1&SVM2&SVM3 |
| --- | --- | --- | --- | --- | --- |
| A_2A_ antagonists | PubChem | 86,844 | 17,687 | 36,869 | 8,073 |
|  | MDDR | 1,470 | 400 | 366 | 238 |
| D_2_ agonists | PubChem | 22,308 | 18,927 | 27,922 | 6,164 |
|  | MDDR | 1,358 | 997 | 1,730 | 486 |
| A_2A_ antagonists &  D_2_ agonists | PubChem | 141 | 18 | 31 | 0 |
|  | MDDR | 2 | 5 | 8 | 0 |
| Total hits | PubChem | 162 (89 passed Rule of 5) | | | |
|  | MDDR | 10 | | | |
