# Supplementary material for: Discovery of indolylpiperazinylpyrimidines with dual-target profiles at adenosine A2A and dopamine D2 receptors for Parkinson's disease treatment
Source: PLoS One. 2018 Jan 5;13(1):e0188212. doi: 10.1371/journal.pone.0188212 (PMC5755735; doi:10.1371/journal.pone.0188212)
Supplement: S3 Table — (DOC) [file pone.0188212.s003.doc]

**S3 Table. The Compound Identification Number (CID) and chemical structures of 172 hits.**

| 1 | 16264012 |  |
| --- | --- | --- |
| 2 | 16542213 |  |
| 3 | 16542200 |  |
| 4 | 16413524 |  |
| 5 | 16413465 |  |
| 6 | 16379184 |  |
| 7 | 16247985 |  |
| 8 | 16247414 |  |
| 9 | 16247251 |  |
| 10 | 16245373 |  |
| 11 | 16082810 |  |
| 12 | 16072539 |  |
| 13 | 16000665 |  |
| 14 | 17967773 |  |
| 15 | 17491036 |  |
| 16 | 17459177 |  |
| 17 | 17451028 |  |
| 18 | 13710945 |  |
| 19 | 13534160 |  |
| 20 | 11675983 |  |
| 21 | 11669387 |  |
| 22 | 15602784 |  |
| 23 | 15147594 |  |
| 24 | 15147591 |  |
| 25 | 13163309 |  |
| 26 | 13157485 |  |
| 27 | 13008197 |  |
| 28 | 12439246 |  |
| 29 | 12394141 |  |
| 30 | 12394138 |  |
| 31 | 12272680 |  |
| 32 | 12074207 |  |
| 33 | 11957288 |  |
| 34 | 11807045 |  |
| 35 | 11784905 |  |
| 36 | 11760941 |  |
| 37 | 10472112 |  |
| 38 | 10434348 |  |
| 39 | 11586584 |  |
| 40 | 11502069 |  |
| 41 | 11499570 |  |
| 42 | 11436381 |  |
| 43 | 11406029 |  |
| 44 | 11356338 |  |
| 45 | 11280247 |  |
| 46 | 11218719 |  |
| 47 | 11211874 |  |
| 48 | 11211873 |  |
| 49 | 11199944 |  |
| 50 | 11188377 |  |
| 51 | 11179094 |  |
| 52 | 11129788 |  |
| 53 | 11117087 |  |
| 54 | 11113212 |  |
| 55 | 11113211 |  |
| 56 | 10990391 |  |
| 57 | 10950355 |  |
| 58 | 10939209 |  |
| 59 | 10928161 |  |
| 60 | 10824197 |  |
| 61 | 10810027 |  |
| 62 | 10810026 |  |
| 63 | 10706218 |  |
| 64 | 10694291 |  |
| 65 | 10682694 |  |
| 66 | 10682693 |  |
| 67 | 10657936 |  |
| 68 | 10643502 |  |
| 69 | 10334610 |  |
| 70 | 10275445 |  |
| 71 | 10252052 |  |
| 72 | 10204026 |  |
| 73 | 10196517 |  |
| 74 | 10027860 |  |
| 75 | 9508606 |  |
| 76 | 7938674 |  |
| 77 | 8850510 |  |
| 78 | 8717516 |  |
| 79 | 8717486 |  |
| 80 | 8717480 |  |
| 81 | 8717478 |  |
| 82 | 8717459 |  |
| 83 | 8717456 |  |
| 84 | 8717455 |  |
| 85 | 8717454 |  |
| 86 | 8717445 |  |
| 87 | 8717438 |  |
| 88 | 8717433 |  |
| 89 | 8717370 |  |
| 90 | 8717369 |  |
| 91 | 8717366 |  |
| 92 | 8717365 |  |
| 93 | 8717316 |  |
| 94 | 8717315 |  |
| 95 | 8717312 |  |
| 96 | 8717311 |  |
| 97 | 8717310 |  |
| 98 | 8717309 |  |
| 99 | 8717308 |  |
| 100 | 8717307 |  |
| 101 | 8717261 |  |
| 102 | 8717251 |  |
| 103 | 8717234 |  |
| 104 | 8717210 |  |
| 105 | 8717208 |  |
| 106 | 8717207 |  |
| 107 | 8717197 |  |
| 108 | 8717168 |  |
| 109 | 8717161 |  |
| 110 | 8717160 |  |
| 111 | 8717157 |  |
| 112 | 8717156 |  |
| 113 | 8717135 |  |
| 114 | 7596323 |  |
| 115 | 7596321 |  |
| 116 | 7437730 |  |
| 117 | 4878637 |  |
| 118 | 6475897 |  |
| 119 | 6475896 |  |
| 120 | 6324665 |  |
| 121 | 6183355 |  |
| 122 | 5842041 |  |
| 123 | 5479268 |  |
| 124 | 5351745 |  |
| 125 | 5329349 |  |
| 126 | 5251092 |  |
| 127 | 4903160 |  |
| 128 | 3613782 |  |
| 129 | 4782319 |  |
| 130 | 3930694 |  |
| 131 | 3794574 |  |
| 132 | 3747930 |  |
| 133 | 3708270 |  |
| 134 | 3558439 |  |
| 135 | 3075503 |  |
| 136 | 3075502 |  |
| 137 | 2919972 |  |
| 138 | 2767322 |  |
| 139 | 2767321 |  |
| 140 | 2670659 |  |
| 141 | 2669568 |  |
| 142 | 2557985 |  |
| 143 | 2455761 |  |
| 144 | 2421978 |  |
| 145 | 1973185 |  |
| 146 | 1608116 |  |
| 147 | 490437 |  |
| 148 | 490434 |  |
| 149 | 437043 |  |
| 150 | 437034 |  |
| 151 | 436136 |  |
| 152 | 418925 |  |
| 153 | 411888 |  |
| 154 | 411759 |  |
| 155 | 411640 |  |
| 156 | 411594 |  |
| 157 | 411093 |  |
| 158 | 410973 |  |
| 159 | 274942 |  |
| 160 | 182566 |  |
| 161 | 113856 |  |
| 162 | 113855 |  |

| 1 | 18907 |  |
| --- | --- | --- |
| 2 | 29326 |  |
| 3 | 29327 |  |
| 4 | 53478 |  |
| 5 | 113106 |  |
| 6 | 121486 |  |
| 7 | 130161 |  |
| 8 | 130170 |  |
| 9 | 133982 |  |
| 10 | 137869 |  |
