# Supplementary material for: Discovery of indolylpiperazinylpyrimidines with dual-target profiles at adenosine A2A and dopamine D2 receptors for Parkinson's disease treatment
Source: PLoS One. 2018 Jan 5;13(1):e0188212. doi: 10.1371/journal.pone.0188212 (PMC5755735; doi:10.1371/journal.pone.0188212)
Supplement: S2 Fig — (DOC) [file pone.0188212.s005.doc]

**Cluster 1:**

1092_47_008 (a2a) 1665_71_013 (a2a)

314_47_test2 (d2) 758_53_12 (d2) 759_53_13 (d2)

**Cluster 2:**

1571_67_007 (a2a) 1663_71_011 (a2a) 1666_71_014 (a2a)

1668_71_016 (a2a)

660_32_32 (d2) 664_32_30e (d2) 653_32_17a (d2)

654_32_17b (d2) 666_32_30g (d2) 230_32_30a (d2)

657_32_29a (d2) 655_32_20a (d2) 656_32_20b (d2) MDDR_221059 (d2)

**Cluster 3:**

1869_59_005 (a2a) 1868_59_004 (a2a) 1867_59_003 (a2a) 1444_62_020 (a2a)

1866_59_002 (a2a)

MDDR_319038 (d2) MDDR_319042 (d2) MDDR_319041 (d2) MDDR_319044 (d2) MDDR_319037(d2)

MDDR_319043 (d2) MDDR_319039 (d2) 528_15_37 (d2) 528_15_38 (d2)

**Cluster 4:**

0065_08_028 (a2a) 029_07_2b (d2) 0318_18_014 (a2a) 1739_18_008 (a2a)

**Cluster 5:**

1779_18_040 (a2a) 1755_18_036 (a2a) 1758_18_012 (a2a)

1756_18_031 (a2a) 1759_18_030 (a2a) 1779_18_040 (a2a)

MDDR_183949 (d2) MDDR_183723 (d2) 747_50_15 (d2) 749_50_17 (d2) 751_50_20 (d2)

**Cluster 6:**

0622_30_032 (a2a) MDDR_341036 (a2a) MDDR_161841 (d2)

**Cluster 7:**

MDDR_318131 (a2a) MDDR_318130 (a2a) MDDR_318132 (a2a) MDDR_289523 (a2a)

MDDR_297311 (d2) 426_07_3b (d2) 425_07_3a (d2)

424_07_1c (d2) 028_07_2a (d2)

**Cluster 8:**

1765_18_046 (a2a) 1741_18_020 (a2a) 0317_18_028 (a2a)

1764_18_016 (a2a) 1145_49_011 (a2a) 0036_05_072 (a2a)

711_41_3d (d2) 264_41_3e (d2) 268_41_3k (d2)

267_41_3h (d2) 266_41_3g (d2) 606_31_59c (d2)

**Cluster 9:**

1670_71_018 (a2a) 1683_03_018 (a2a) 1583_67_023 (a2a)

1570_67­_006 (a2a)

584_30_40 (d2)

**Cluster 10:**

0500_27_063 (a2a) 0501_27_065 (a2a) 0518_27_064 (a2a)

0519_27_066 (a2a) 0510_27_083 (a2a) 0528_27_084 (a2a)

668_33_11 (d2) MDDR_190847 (d2) 671_33_12d (d2)

670_33_12c (d2) 669_33_12a (d2) 241_33_12b (d2)

**S2 Fig.** Ten clusters from dendrogram and cluster analysis.
