# Supplementary material for: Discovery of indolylpiperazinylpyrimidines with dual-target profiles at adenosine A2A and dopamine D2 receptors for Parkinson's disease treatment
Source: PLoS One. 2018 Jan 5;13(1):e0188212. doi: 10.1371/journal.pone.0188212 (PMC5755735; doi:10.1371/journal.pone.0188212)
Supplement: S6 Fig — (DOC) [file pone.0188212.s009.doc]

**S6 Fig.** Design from cluster 8.
