# Supplementary material for: Discovery of indolylpiperazinylpyrimidines with dual-target profiles at adenosine A2A and dopamine D2 receptors for Parkinson's disease treatment
Source: PLoS One. 2018 Jan 5;13(1):e0188212. doi: 10.1371/journal.pone.0188212 (PMC5755735; doi:10.1371/journal.pone.0188212)
Supplement: S8 Fig — (DOC) [file pone.0188212.s011.doc]

**S8 Fig.** Ten compounds that at least pass one model of A_2A_ and one model of D_2_.
