# Supplementary material for: Discovery of indolylpiperazinylpyrimidines with dual-target profiles at adenosine A2A and dopamine D2 receptors for Parkinson's disease treatment
Source: PLoS One. 2018 Jan 5;13(1):e0188212. doi: 10.1371/journal.pone.0188212 (PMC5755735; doi:10.1371/journal.pone.0188212)
Supplement: S9 Fig — Number of His+-revertant colonies grown on the agar plates containing various chemicals and (A) TA98 strain without “S9 mix”, (B) TA98 strain with “S9 mix”, (C) TA100 strain without “S9 mix”, and (D) TA100 strain with “S9 mix”. Blank indicates spontaneously induced revertants without treatment with any drug or solvent. (DOC) [file pone.0188212.s012.doc]

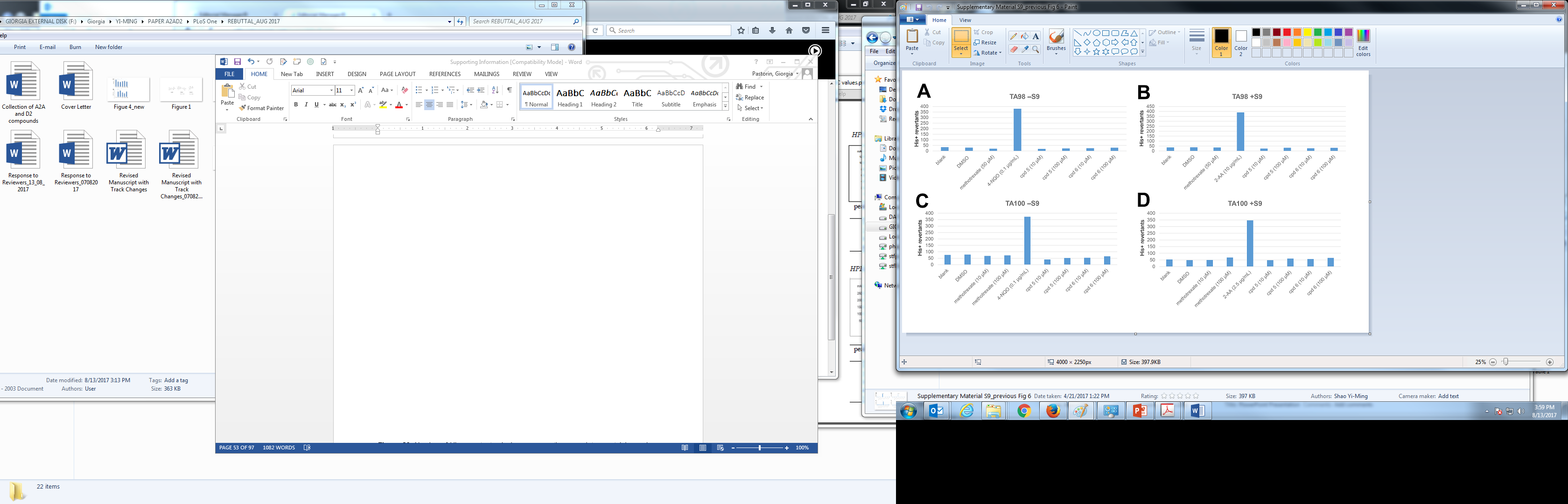


**S9 Fig.**: Number of His^+^-revertant colonies grown on the agar plates containing various chemicals and (A) TA98 strain without “S9 mix”, (B) TA98 strain with “S9 mix”, (C) TA100 strain without “S9 mix”, and (D) TA100 strain with “S9 mix”. Blank indicates spontaneously induced revertants without treatment with any drug or solvent.
