# Supplementary material for: Discovery of indolylpiperazinylpyrimidines with dual-target profiles at adenosine A2A and dopamine D2 receptors for Parkinson's disease treatment
Source: PLoS One. 2018 Jan 5;13(1):e0188212. doi: 10.1371/journal.pone.0188212 (PMC5755735; doi:10.1371/journal.pone.0188212)
Supplement: S10 Fig — Cell viability after treatment of TAMH (A-C) and HL-1 (D-F) cells with positive controls and compounds. (DOC) [file pone.0188212.s013.doc]

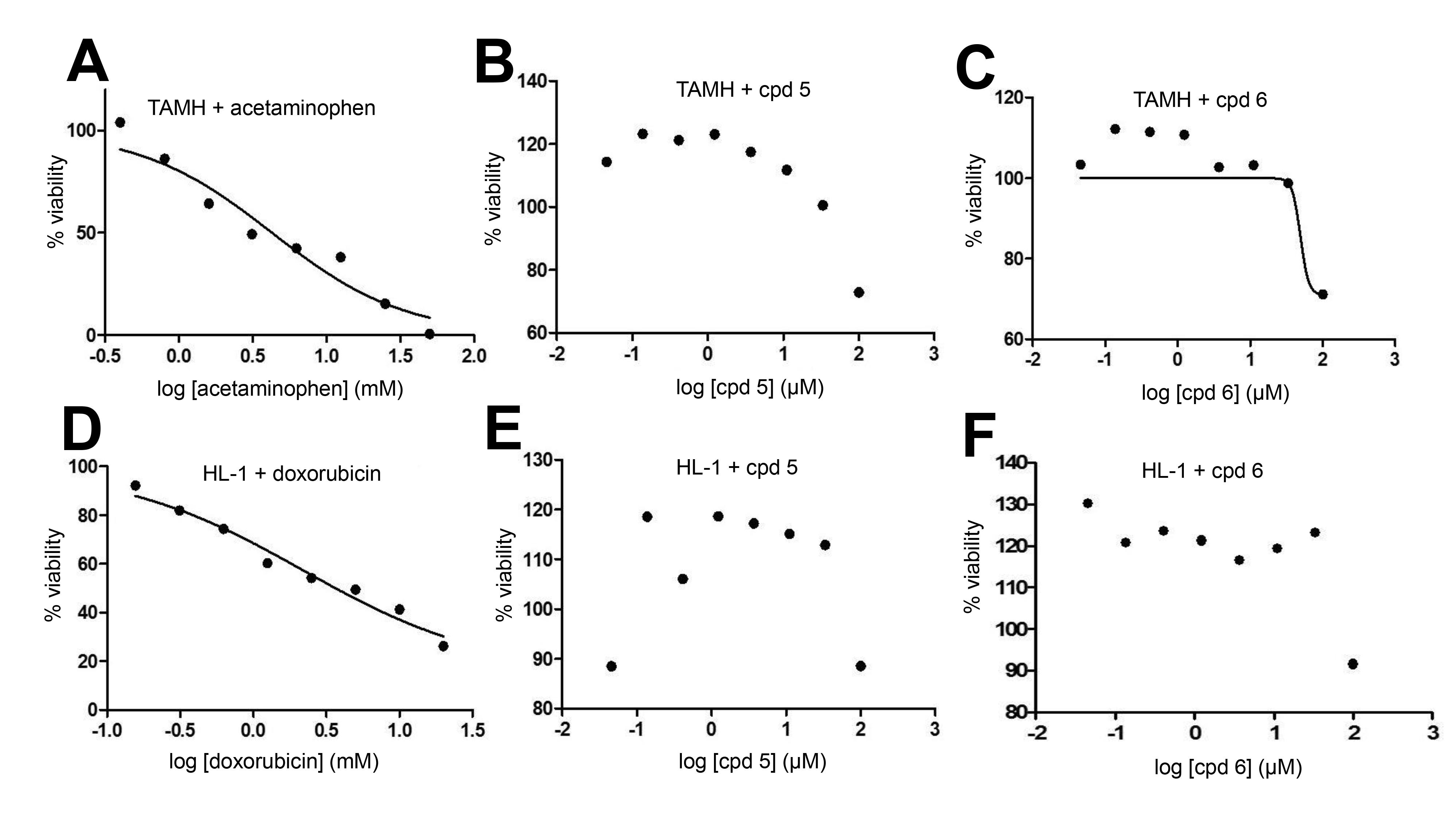


**S10 Fig.**: Cell viability after treatment of TAMH (A-C) and HL-1 (D-F) cells with positive controls and compounds.
